# Egg and Dietary Cholesterol Intake and Risk of All-Cause, Cardiovascular, and Cancer Mortality: A Systematic Review and Dose-Response Meta-Analysis of Prospective Cohort Studies

**DOI:** 10.3389/fnut.2022.878979

**Published:** 2022-05-27

**Authors:** Manije Darooghegi Mofrad, Sina Naghshi, Keyhan Lotfi, Joseph Beyene, Elina Hypponen, Aliyar Pirouzi, Omid Sadeghi

**Affiliations:** ^1^Department of Community Nutrition, School of Nutritional Science and Dietetics, Tehran University of Medical Sciences, Tehran, Iran; ^2^Department of Clinical Nutrition, School of Nutritional Science and Dietetics, Tehran University of Medical Sciences, Tehran, Iran; ^3^Department of Health Research Methods, Evidence, and Impact, McMaster University, Hamilton, ON, Canada; ^4^Chanchlani Research Centre, McMaster University, Hamilton, ON, Canada; ^5^Australian Centre for Precision Health, Unit of Clinical and Health Sciences, University of South Australia, Adelaide, SA, Australia; ^6^South Australian Health and Medical Research Institute, Adelaide, SA, Australia; ^7^Cellular and Molecular Department, Gerash University of Medical Sciences, Gerash, Iran; ^8^Food Security Research Center, Isfahan University of Medical Sciences, Isfahan, Iran; ^9^Department of Community Nutrition, School of Nutrition and Food Science, Isfahan University of Medical Sciences, Isfahan, Iran

**Keywords:** egg, dietary cholesterol, mortality, meta-analysis, dose-response

## Abstract

**Objective:**

This systematic review and meta-analysis of prospective cohort studies examined the associations between egg and dietary cholesterol intake and the risk of mortality from all causes, including cardiovascular disease (CVD) and cancer.

**Methods:**

We searched PubMed, Scopus, ISI Web of Knowledge, and Google Scholar until April 2021, as well as references to the relevant articles retrieved. Random-effects models were used to calculate summary relative risk (RR) and 95% confidence intervals (CIs) for the highest vs. lowest categories of egg and dietary cholesterol intake. Also, linear and non-linear dose–response analyses were conducted to examine the dose-response relationships.

**Results:**

We included 55 studies, comprising data from 2,772,486 individuals with 228,425, 71,745, and 67,211 cases of all-cause, CVD, and cancer mortality, respectively. Intake of each additional egg per day was associated with a 7% higher risk of all-cause (1.07, 95% CI: 1.02–1.12, I^2^ = 84.8%) and a 13% higher risk of cancer mortality (1.13, 95% CI: 1.06–1.20, I^2^ = 54.2%), but was not associated with CVD mortality (1.00, 95% CI: 0.92–1.09, I^2^ = 81.5%). Non-linear analyses showed increased risks for egg consumption of more than 1.5 and 0.5 eggs/day, respectively. Each 100 mg/day increment in dietary cholesterol intake was associated with a 6% higher risk of all-cause mortality (1.06, 95% CI: 1.03–1.08, I^2^ = 34.5%) and a 6% higher risk of cancer mortality (1.06, 95% CI: 1.05–1.07, I^2^ = 0%), but was not associated with CVD mortality (1.04, 95% CI: 0.99–1.10, I^2^ = 85.9%). Non-linear analyses demonstrated elevated risks of CVD and cancer mortality for intakes more than 450 and 250 mg/day, respectively.

**Conclusions and Relevance:**

High-dietary intake of eggs and cholesterol was associated with all-cause and cancer mortality. Little evidence for elevated risks was seen for intakes below 0.5 egg/day or 250 mg/day of dietary cholesterol. Our findings should be considered with caution because of small risk estimates and moderate between-study heterogeneity.

**Systematic Review Registration:**

https://www.crd.york.ac.uk/prospero/display_record.php?RecordID=252564, PROSPERO, identifier: CRD42021252564.

## Introduction

Cardiovascular disease (CVD) and cancer led to 26.9 million deaths worldwide in 2016 (1). The effects of dietary cholesterol and egg intake, as a part of the usual diet, on disease risk and longevity have been debated for decades. Eggs contain essential amino acids, B vitamins, unsaturated fatty acids, choline, lutein, and zeaxanthin (2), some of which (e.g., choline) have CVD-protective effects. However, eggs are also a rich source of dietary cholesterol (186 mg of cholesterol per egg) (3). It contributed to 25% of the total dietary cholesterol consumption, which is known to adversely affect blood lipids and increase the risk of mortality from CVDs.

Studies have also reported a positive link between dietary cholesterol intake and the risk of some cancers (4, 5), although this is less well established. The overall health effects are likely to reflect the interactions of cholesterol with other nutrients in eggs and explain the conflicting results reported for the association between egg consumption and health outcomes. Although findings from randomized clinical trials have suggested a modest contribution of dietary cholesterol or eggs to blood lipids (6). Their generalization to the whole population is questionable due to their wellknown limitations (7). Therefore, findings from prospective cohort studies can provide complementary evidence. Prospective cohort studies on the association between dietary cholesterol and egg intake and mortality have generated conflicting findings. Some studies revealed a possible positive association between egg consumption and mortality risk (8, 9), while others found an inverse (10, 11) or no association (12, 13). Such mixed results have also been reported for dietary cholesterol intake (1, 14, 15).

Two recent meta-analyses showed no significant association between egg consumption and the risk of all-cause and coronary heart disease (CHD) mortality (13, 16). However, these reviews missed several eligible cohort studies (17–20). Moreover, a large number of relevant articles have been published since the release of those meta-analyses (1, 14, 15, 21–26). Those published meta-analyses did not assess the risk of total CVD and cancer mortality and also the dose–response association between egg consumption and mortality.

The inconsistency in the available evidence has resulted in changes to dietary guidelines for cholesterol consumption. For example, until 2015, the American Heart Association Guidelines recommended limiting dietary cholesterol to less than 300 mg/day (27), while the current guidelines have no limitation for cholesterol consumption (28). Overall, a comprehensive systematic review and meta-analysis are needed to summarize available findings on the associations between egg and dietary cholesterol intake from all food sources (e.g., eggs, red meat, poultry, fish, and dairy products) and the risk of mortality. Therefore, we conducted a comprehensive systematic review and dose–response meta-analysis of prospective cohort studies to assess the associations of egg and dietary cholesterol intake with the risk of mortality from all-causes, CVD, and cancer in adults.

## Methods

This systematic review and meta-analysis were conducted in accordance with the Meta-analyses of Observational Studies in Epidemiology (MOOSE) guidelines (29).

### Search Strategy

We searched online databases, including PubMed, Scopus, ISI Web of Science, Embase, and Google Scholar, up to April 2021 (Supplementary Table 1). The search was done without applying any filters, including publication date or the language of articles. In addition, the reference lists of the selected articles and recent reviews were cross-checked to identify any articles that may have been missed.

### Inclusion Criteria

Studies were selected if they (1) used a prospective observational design; (2) were conducted on an adult population (≥18 years); and (3) reported relative effect estimates, including risk ratio (RR), hazard ratio (HR), and odds ratio (OR), with 95% confidence intervals (CI), to determine the associations of egg and dietary cholesterol intake with the risk of all-cause, cancer, or CVD mortality. If the results from one study were published in more than one article, we selected the most recent one; otherwise, the one with the greatest number of cases or with the highest quality was included.

### Exclusion Criteria

We excluded studies if they (1) were conducted on subjects who had CVD or cancer at baseline; (2) were letters, abstracts, unpublished studies, reviews, comments, ecological studies, or meta-analyses; and (3) had insufficient data for systematic review and meta-analysis.

### Data Extraction

Two investigators (MDM and SN) screened and extracted data independently, and another author (KL) checked them for accuracy. The following items were extracted from each eligible study: first author's name, study location, gender, age, sample size, number of cases, study period, categories of egg and dietary cholesterol intake, methods used for dietary evaluation, relative risks, and 95% confidence intervals, and variables adjusted for in the analysis. If an included study reported several effect estimates, the one that was controlled for the most confounding variables was used for the meta-analysis.

### Risk of Bias Assessment

To assess the risk of bias among included studies, we used the risk of bias in non-randomized studies of exposures tool. This tool comprises seven domains through which bias might be introduced. The questions in these domains include bias due to confounding, bias in the selection of participants in the study, bias in the classification of exposures, bias due to departure from intended exposures, bias due to missing data, bias in the measurement of outcomes, and bias in the selection of reported results. Under each domain, we categorized studies as having a low, moderate, serious, or critical risk of bias.

### Statistical Methods

We included the RRs (and 95% CIs) of mortality for the comparison between the highest and lowest intakes of eggs and dietary cholesterol in the meta-analysis. The risk estimates were combined using a random-effects model. If a study reported subgroup risk estimates stratified by gender or any other variables, we first pooled the subgroup estimates using a fixed-effects model, and then the pooled risk estimate was included in the main meta-analysis. In addition, if a study presented the RRs for deaths due to different types of CVDs or cancers, not for overall outcomes, we first pooled the RRs using a fixed-effects model, and then the pooled RR of CVD or cancer mortality was included in the meta-analysis.

Cochran's Q test and the I^2^ statistic were applied to evaluate heterogeneity among studies. Subgroup and meta-regression analyses were conducted to detect possible sources of heterogeneity. Publication bias was assessed using Egger's linear regression test (30). In the case of substantial publication bias, the trim-and-fill method was applied to detect the effect of probable missing studies on the overall RR (31). To assess the dependency of the overall RR on one study, sensitivity analysis was conducted using a random-effects model.

A linear dose–response analysis was conducted using the generalized least squares trend estimation method, described by Greenland and Longnecker (32) and Orsini et al. (33). Estimated study-specific slopes were combined using a random-effects model to provide an overall average slope. In the non-linear dose–response analysis, exposures were modeled using restricted cubic splines with three knots at percentiles of 10, 50, and 90% of the distribution. The correlation within each set of provided risk estimates was considered, and the study-specific estimates were combined using a one-stage linear mixed-effects meta-analysis. The significance level for non-linearity was assessed by testing whether or not the coefficient of the second spline was equal to zero. All analyses were done using STATA version 16.0. *P* < 0.05 was assumed to be statistically significant for all tests.

## Results

**Figure 1:**
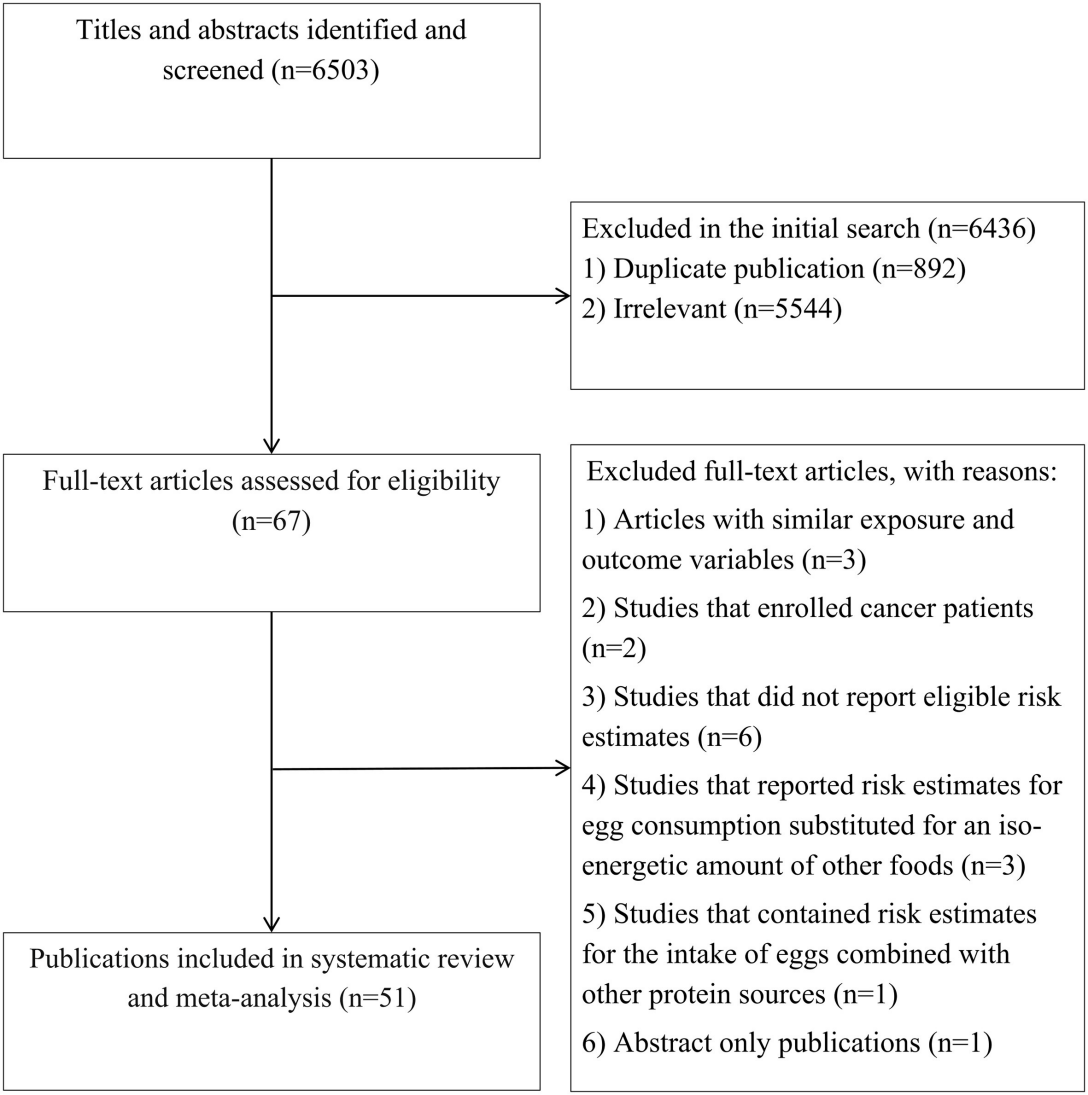
Flow diagram of study selection.

In total, we identified 6,503 articles in our initial search. After excluding duplicate articles and studies that did not meet the inclusion criteria, there were 67 potentially relevant publications (Figure 1). After full-text reviews, we excluded two articles owing to enrolling cancer patients (34, 35). Six publications were excluded because they did not report eligible risk estimates (36–41). Three articles reported risk estimates for egg consumption substituted for an iso-energetic amount of other foods and thus were excluded (42–44). One study was excluded because it contained risk estimates for the intake of eggs combined with other protein sources (45). We also excluded one abstract without the required data for a meta-analysis (46). In addition, we found studies with significant participant overlap, including articles from the National Health and Nutrition Examination Survey (NHANES) (16, 22), the Health Professionals Follow-Up Study (HPFS) (47, 48), and the China Health and Nutrition Survey (CHNS) (14, 49). Since these studies reported risk estimates for similar exposure and outcome variables, we included only the one with higher quality or with the highest number of cases and excluded the duplicate publications (16, 47, 49). The articles by Sun et al. (21) and Chen et al. (24) used data from the Women's Health Initiative study, with more complete data presented by Sun et al. However, the study by Chen et al. was included because it presented the RR in relation to both dietary cholesterol and egg consumption. Dehghan et al. (1) used three different datasets; two with CVD patients and one with the general population. Therefore, we included the risk estimates reported for the general population and excluded those related to patients with vascular diseases. In total, 55 studies (51 publications) remained for the final meta-analysis (1, 8–15, 17–26, 48, 50–80), of which 25 studies examined the association of egg consumption with all-cause mortality (1, 8, 9, 12–15, 17–23, 25, 26, 53, 57, 65, 68, 74–76, 79, 80), 21 with CVD mortality (1, 8–13, 15, 18, 19, 21, 22, 25, 26, 56, 64, 65, 71, 73, 75, 76), and 19 with cancer mortality (9, 12, 15, 19, 21, 25, 26, 48, 52, 59, 61–63, 65, 66, 69, 75–78). In terms of dietary cholesterol intake, 11 publications reported risk estimates for all-cause mortality (1, 8, 14, 15, 18, 22, 24, 54, 55, 58, 60), 10 for CVD mortality (1, 8, 15, 18, 22, 24, 50, 67, 70, 72), and 3 for cancer mortality (15, 24, 51).

### Characteristics of the Included Studies

Supplementary Tables 2–4 show the general characteristics of studies included in the current systematic review and meta-analysis. The number of participants in these studies ranged from 162 to 521,120 individuals with an age range between 15 and 103 years. In total, 2,772,486 participants were included in the 55 studies we considered. During the follow-up periods ranging from 5 to 32 years, 228,425 participants died from all causes, 71,745 from CVD, and 67,211 from cancer. Also, nine included only men (48, 50, 52, 53, 57, 58, 67, 74, 79), six included only women (9, 21, 24, 51, 69, 72), and five publications reported risk estimates for men and women separately (61, 65, 71, 77, 78). A total of 13 studies were from the United States (15, 17, 21, 22, 24, 50–54, 56, 58, 63, 66, 68, 71), 12 were from Europe (12, 18, 25, 26, 55, 57, 58, 67, 73, 74, 76, 79, 80), 17 were from Asia (9–11, 13, 14, 19, 20, 23, 58, 59, 61, 62, 65, 69, 70, 75, 77, 78), and four publications (13 studies) describe studies that recruited populations from more than one country (1, 8, 48, 60). Dietary intakes were assessed using food frequency questionnaires in 43 publications (1, 8–11, 13, 15, 17–21, 23–26, 48, 50–53, 55–57, 59, 61–69, 71–73, 75–78), 6 applied to food recall or record (14, 22, 54, 58, 70, 74), and 3 used dietary history (12, 60, 79). Based on the ROBINS-E tool, 24 studies (25%) were rated as having a serious risk of bias and 27 studies (75%) a moderate risk of bias (Supplementary Table 5).

### Findings From the Meta-Analysis on All-Cause Mortality

#### Egg Consumption and All-Cause Mortality

A total of 27 studies (22 publications) (1, 8, 9, 12–15, 17–23, 25, 26, 53, 57, 65, 68, 74, 79) examined the association between egg consumption and risk of all-cause mortality by comparing the highest and lowest egg intakes. In these studies, the median egg intake classified as “high” was 1.07 eggs/day (IQR: 0.73–1.24) and 0.03 egg/day (IQR: 0–0.07) as “low,” with one study not providing quantification (20). These studies included a total of 1,153,367 participants and recorded 226,990 deaths from all causes. Combining RRs from these studies did not provide evidence for an association between egg consumption and all-cause mortality (Pooled RR: 1.03, 95% CI: 0.97–1.09, *I*^2^ = 85.3%, *P*_heterogeneity_ < 0.001) (Table 1; Supplementary Figure 1).

**Table 1:** Summary risk estimates for the associations between egg and cholesterol intake and risk of all-cause mortality in adults aged ≥18 years^*a*^.

	**#RR^**b**^**	**Pooled RR (95% CI)^**c**^**	**I^**2**^ (%)^**d**^**	***P*-heterogeneity^**e**^**	***P*-meta-regression**
**The highest vs. lowest comparison**					
Egg intake					
Overall	22	1.03 (0.97–1.09)	85.3	<0.001	
Subgroup analysis					
Study location					
US	7	1.13 (1.09–1.016)	43.9	0.09	0.05
Non-US	15	0.98 (0.90–1.06)	74.3	<0.001	
Gender					
Both	16	1.01 (0.94–1.08)	88.1	<0.001	0.42
Male	4	1.07 (0.89–1.29)	71.5	0.01	
Female	2	1.43 (0.79–2.56)	75.2	0.04	
Follow-up duration					
≥15 years	11	1.06 (0.99–1.13)	84.4	<0.001	0.46
<15 years	11	1.00 (0.92–1.08)	66.9	0.001	
Dietary assessment tools					
FFQ or DHQ	18	1.06 (1.00–1.12)	83.2	<0.001	0.05
Food recall or record	4	0.89 (0.72–1.11)	79.9	0.002	
Adjustment for energy					
Yes	13	1.01 (0.95–1.08)	86.2	<0.001	0.49
No	9	1.07 (0.96–1.21)	81.3	<0.001	
Adjustment for BMI					
Yes	17	1.03 (0.96–1.10)	88	<0.001	0.80
No	5	1.05 (0.98–1.13)	30.6	0.21	
Adjustment for lipid-lowering medication					
Yes	4	1.01 (0.84–1.22)	84.1	<0.001	0.78
No	18	1.04 (0.98–1.10)	84.9	<0.001	
Dietary cholesterol intake					
Overall	9	1.07 (1.02–1.13)	47.8	0.05	
Subgroup analysis					
Study location					
US	4	1.13 (1.10–1.16)	0	0.49	0.02
Non-US	5	1.00 (0.94–1.07)	0	0.74	
Gender					
Both	8	1.06 (0.99–1.13)	52.5	0.04	0.70
Male	-	–	–	–	
Female	1	1.09 (1.03–1.16)	–	–	
Follow-up duration					
≥15 years	4	1.09 (1.03–1.16)	61.1	0.05	0.27
<15 years	5	1.03 (0.96–1.09)	0	0.96	
Dietary assessment tools					
FFQ or DHQ	7	1.09 (1.04–1.14)	39.9	0.12	0.24
Food recall or record	2	0.98 (0.82–1.17)	45.2	0.17	
Adjustment for energy					
Yes	6	1.09 (1.04–1.15)	43.6	0.11	0.18
No	3	1.01 (0.93–1.09)	0	0.95	
Adjustment for BMI					
Yes	5	1.06 (0.97–1.15)	69.8	0.01	0.96
No	4	1.08 (1.02–1.14)	0	0.89	
Adjustment for lipid-lowering medication					
Yes	1	1.09 (0.95–1.26)	–	–	0.81
No	8	1.07 (1.01–1.13)	54.1	0.03	
**Linear dose-response association**					
Egg intake (per 1 egg/d increase)					
Overall	24	1.07 (1.02–1.12)	84.8	<0.001	
Subgroup analysis					
Study location					
US	7	1.13 (1.10–1.17)	47.4	0.07	0.27
Non-US	17	1.04 (0.96–1.13)	82.5	<0.001	
Gender					
Both	18	1.05 (0.99–1.12)	87.1	<0.001	0.65
Male	4	1.08 (0.92–1.25)	68.9	0.02	
Female	2	1.15 (1.12–1.19)	0	0.45	
Follow-up duration					
≥15 years	12	1.06 (1.01–1.12)	84.8	<0.001	0.33
<15 years	12	1.08 (0.98–1.19)	82.3	<0.001	
Dietary assessment tools					
FFQ or DHQ	20	1.09 (1.04–1.14)	83.3	<0.001	0.16
Food recall or record	4	0.95 (0.84–1.09)	64.2	0.03	
Adjustment for energy					
Yes	13	1.07 (1.01–1.13)	86.7	<0.001	0.84
No	11	1.07 (0.99–1.16)	77.4	<0.001	
Adjustment for BMI					
Yes	18	1.05 (1.00–1.11)	85.4	<0.001	0.32
No	6	1.14 (0.99–1.33)	85.8	<0.001	
Adjustment for lipid-lowering medication					
Yes	5	1.07 (0.90–1.27)	83.5	<0.001	0.90
No	19	1.07 (1.02–1.13)	85.8	<0.001	
Dietary cholesterol intake (per 100 mg/d increase)					
Overall	8	1.06 (1.03–1.08)	34.5	0.15	
Subgroup analysis					
Study location					
US	4	1.06 (1.03–1.10)	68	0.02	0.38
Non-US	4	1.01 (0.93–1.11)	0	0.88	
Gender					
Both	6	1.05 (1.04–1.06)	0	0.86	0.29
Male	1	1.02 (0.93–1.12)	–	–	
Female	1	1.15 (1.08–1.22)	–	–	
Follow-up duration					
≥15 years	5	1.06 (1.03–1.09)	57.5	0.05	0.06
<15 years	3	0.98 (0.79–1.20)	0	0.68	
Dietary assessment tools					
FFQ or DHQ	5	1.06 (1.04–1.09)	53.7	0.07	0.16
Food recall or record	3	1.00 (0.92–1.10)	0	0.63	
Adjustment for energy					
Yes	7	1.06 (1.03–1.08)	42.5	0.10	0.63
No	1	0.89 (0.46–1.73)	–	–	
Adjustment for BMI					
Yes	5	1.05 (1.04–1.06)	0	0.74	0.02
No	3	1.14 (1.08–1.21)	0	0.67	
Adjustment for lipid-lowering medication					
Yes	1	1.05 (1.03–1.08)	–	–	0.98
No	7	1.06 (1.01–1.12)	43.7	0.09	

a *BMI, body mass index; CI, confidence interval; RR, relative risk; DHQ, dietary history questionnaire; FFQ, food frequency questionnaire; US, United States*.

b *Number of risk estimates*.

c *Obtained from the random-effects model*.

d *Inconsistency, the percentage of variation across studies due to heterogeneity*.

e *Obtained from the Q-test*.

**Figure 2:**
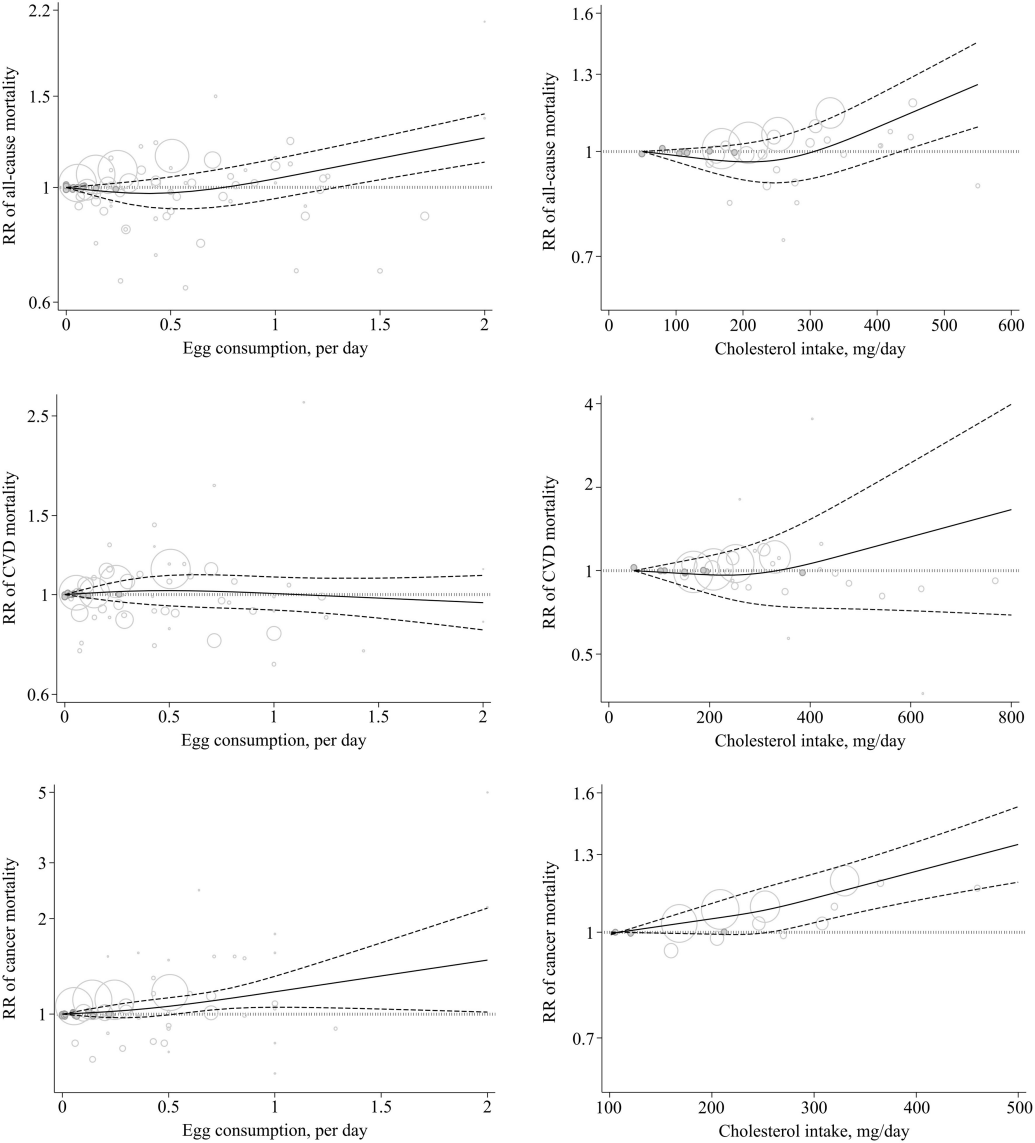
Non-linear dose–response association of egg (based on egg/day) and cholesterol (based on mg/day) intake with the risk of mortality from all-cause, CVD, and cancer in adults aged ≥18 years. Solid line indicates the spline model. Dashed line presents the 95% CI. CVD, cardiovascular disease; RR, relative risk; CI, confidence interval.

A total of 28 studies (23 publications) (1, 8, 9, 13–15, 17–23, 25, 26, 53, 57, 65, 68, 74–76, 80) were identified for inclusion in the dose–response analysis. Each additional egg per day was associated with a 7% higher risk of all-cause mortality (Pooled RR: 1.07, 95% CI: 1.02–1.12, *I*^2^ = 84.8%, *P*_heterogeneity_ < 0.001) (Table 1; Supplementary Figure 2). There was evidence for a non-linear association (*P*_non-*linearity*_ = 0.003); egg consumption from the lowest amount to one egg per day had no association with all-cause mortality, while the consumption of more than 1 egg per day (~1.5 eggs/day) was associated with an increase in all-cause mortality (Figure 2; Supplementary Table 6).

#### Dietary Cholesterol Intake and All-Cause Mortality

A total of 14 cohort studies (9 publications) (1, 8, 14, 15, 18, 22, 24, 55, 60), including a total of 172,147 deaths among 852,279 participants, investigated the association between high vs. low cholesterol intake and all-cause mortality. In these studies, the median for high cholesterol intake was 419 mg/day (IQR: 330–453) and the median for low cholesterol intake was 114 mg/day (IQR: 80–147). However, two studies provided no quantification (55, 60). The summary RR was 1.07 (95% confidence interval: 1.02–1.13, *I*^2^ = 47.8%, *P*_heterogeneity_ = 0.05) (Table 1; Supplementary Figure 3).

In the dose–response meta-analysis, including 14 studies (1, 8, 14, 15, 18, 22, 24, 54, 58), we found that a 100 mg/day increase in dietary cholesterol intake was associated with a 6% greater all-cause mortality (pooled RR: 1.06, 95% CI: 1.03–1.08, *I*^2^ = 34.5%, *P*_heterogeneity_ = 0.16) (Table 1; Supplementary Figure 4). There was evidence of a non-linear association (*P*_non-*linearity*_ < 0.001) so that the risk of all-cause mortality increased from 450 mg/day dietary cholesterol to the higher amounts (Figure 2; Supplementary Table 7).

### Findings From the Meta-Analysis on CVD Mortality

#### Egg Consumption and CVD Mortality

We included 16 cohort studies (1, 9–13, 15, 18, 19, 21, 22, 25, 26, 56, 65, 71) with 1,479,181 participants and 69,325 cases of deaths in the analysis of the highest vs. lowest egg consumption and CVD mortality. In this analysis, the highest and lowest intakes of eggs were defined as median intakes of 1 egg/day (IQR: 0.7–1.24) and 0.007 egg/day (IQR: 0–0.06), respectively. The summary RR for CVD mortality in relation to egg consumption was 1.01 (95% CI: 0.90–1.13, *I*^2^ = 83.1%; *P*_heterogeneity_ < 0.001) (Table 2; Supplementary Figure 5).

**Table 2:** Summary risk estimates for the associations between egg and cholesterol intake and risk of CVD mortality in adults aged ≥18 years^*a*^.

	**#RR^**b**^**	**Pooled RR (95% CI)^**c**^**	**I^**2**^ (%)^**d**^**	***P*-heterogeneity^**e**^**	***P*-meta-regression**
**The highest vs. lowest comparison**					
Egg intake					
Overall	16	1.01 (0.90–1.13)	83.1	<0.001	
Subgroup analysis					
Study location					
US	5	1.15 (1.05–1.25)	50.1	0.09	0.23
Non-US	11	0.97 (0.85–1.10)	60.6	0.005	
Gender					
Both	14	0.98 (0.87–1.12)	83.4	<0.001	0.16
Male	0	–	–	–	
Female	2	1.24 (1.14–1.34)	0	0.89	
Follow-up duration					
≥15 years	5	1.11 (0.99–1.24)	70.3	0.009	0.11
<15 years	11	0.96 (0.85–1.08)	51	0.02	
Dietary assessment tools					
FFQ or DHQ	15	1.02 (0.91–1.14)	83.9	<0.001	0.61
Food recall or record	1	0.89 (0.64–1.23)	–	–	
Adjustment for energy					
Yes	11	1.07 (0.98–1.16)	60.4	0.005	0.008
No	5	0.91 (0.67–1.24)	57.7	0.05	
Adjustment for BMI					
Yes	13	0.99 (0.87–1.12)	85.4	<0.001	0.48
No	3	1.16 (0.82–1.64)	64.4	0.06	
Adjustment for lipid-lowering medication					
Yes	3	0.93 (0.80–1.07)	0	0.91	0.68
No	13	1.02 (0.90–1.15)	85.6	<0.001	
Dietary cholesterol intake					
Overall	9	1.09 (0.96–1.24)	64.1	0.004	
Subgroup analysis					
Study location					
US	5	1.13 (1.08–1.18)	0	0.75	0.27
Non-US	4	0.99 (0.62–1.59)	82	0.001	
Gender					
Both	5	1.05 (0.78–1.39)	77.7	0.001	0.97
Male	2	1.01 (0.77–1.33)	26.8	0.24	
Female	2	1.20 (1.07–1.34)	0	0.65	
Follow-up duration					
≥15 years	2	1.13 (1.08–1.18)	0	0.32	0.28
<15 years	7	1.06 (0.81–1.40)	68.4	0.004	
Dietary assessment tools					
FFQ or DHQ	7	1.13 (1.01–1.26)	54.3	0.04	0.30
Food recall or record	2	0.64 (0.23–1.74)	83	0.01	
Adjustment for energy					
Yes	8	1.07 (0.96–1.19)	51.9	0.04	0.09
No	1	3.53 (1.57–7.95)	–	–	
Adjustment for BMI					
Yes	6	1.02 (0.84–1.24)	58.6	0.03	0.42
No	3	1.27 (0.90–1.78)	78.7	0.009	
Adjustment for lipid lowering medication					
Yes	0	–	–	–	-
No	9	1.09 (0.96–1.24)	64.1	0.004	
**Linear dose-response association**					
Egg intake (per 1 egg/d increase)					
Overall	21	1.00 (0.92– 1.09)	81.6	<0.001	
Subgroup analysis					
Study location					
US	15	0.96 (0.85– 1.09)	72.6	<0.001	0.21
Non-US	6	1.17 (1.11– 1.23)	31	0.20	
Gender					
Both	19	0.99 (0.89– 1.09)	81.9	<0.001	0.60
Male	–	–		–	
Female	2	1.10 (0.84– 1.43)	66.2	0.08	
Follow-up duration					
≥10 years	8	1.05 (0.97– 1.15)	78.2	<0.001	0.74
<10 years	13	1.00 (0.84– 1.19)	74.4	<0.001	
Dietary assessment tools					
FFQ or DHQ	20	1.00 (0.92– 1.10)	82.1	<0.001	0.63
Food recall or record	1	0.86 (0.61– 1.21)	–	–	
Adjustment for energy					
Yes	13	1.08 (1.00– 1.15)	63.6	0.001	0.27
No	8	0.98 (0.78– 1.22)	81.3	<0.001	
Adjustment for BMI					
Yes	17	0.97 (0.89– 1.07)	81.2	<0.001	0.19
No	4	1.44 (0.94– 2.18)	83.4	<0.001	
Adjustment for lipid-lowering medication					
Yes	5	1.11 (0.78–1.58)	76.4	0.002	0.68
No	16	0.99 (0.90–1.08)	83.6	<0.001	
Dietary cholesterol intake (per 100 mg/d increase)					
Overall	9	1.04 (0.99– 1.10)	85.9	<0.001	
Subgroup analysis					
Study location					
US	5	1.10 (1.03– 1.18)	77.6	0.001	0.32
Non-US	4	0.99 (0.91– 1.08)	83	0.001	
Gender					
Both	6	1.02 (0.96– 1.09)	81.4	<0.001	0.91
Male	2	0.98 (0.95– 1.01)	0	0.57	
Female	1	1.29 (1.17– 1.43)	–	–	
Follow-up duration					
≥10 years	3	1.11 (1.02– 1.20)	88.7	<0.001	0.31
<10 years	6	1.00 (0.92– 1.08)	72.9	0.002	
Dietary assessment tools					
FFQ or DHQ	7	1.07 (1.01– 1.12)	86.7	<0.001	0.18
Food recall or record	2	0.92 (0.74– 1.15)	56.8	0.12	
Adjustment for energy					
Yes	8	1.03 (0.98– 1.08)	85.5	<0.001	0.10
No	1	1.38 (1.13– 1.68)	–	–	
Adjustment for BMI					
Yes	6	1.01 (0.96– 1.06)	80.8	<0.001	0.13
No	3	1.19 (0.95– 1.50)	93.3	<0.001	
Adjustment for lipid-lowering medication					
Yes	1	1.07 (1.02–1.12)	87.1	<0.001	0.96
No	8	1.04 (0.98–1.11)	–	–	

a *BMI, body mass index; CI, confidence interval; RR, relative risk; DHQ, dietary history questionnaire; FFQ, food frequency questionnaire; US, United States*.

b *Number of risk estimates*.

c *Obtained from the random-effects model*.

d *Inconsistency, the percentage of variation across studies due to heterogeneity*.

e *Obtained from the Q-test*.

A total of 26 cohort studies (21 publications) (1, 8–13, 15, 18, 19, 21, 22, 25, 26, 56, 64, 65, 71, 73, 75, 76) were included in the dose–response analysis. The summary RR for CVD mortality based on 1 egg/day increase was 1.00 (95% CI: 0.92–1.09, *I*^2^ = 81.5%, *P*_heterogeneity_ < 0.001) (Table 2; Supplementary Figure 6). There was no evidence of a non-linear association (*P*_non-*linearity*_ = 0.43) (Figure 2; Supplementary Table 6).

#### Dietary Cholesterol Intake and CVD Mortality

A total of nine prospective studies (1, 15, 18, 22, 24, 50, 67, 70, 72) were included in the analysis of the highest (median: 420 mg/day, IQR: 348–580) vs. lowest (median: 149 mg/day, IQR: 111–187) intake of dietary cholesterol and CVD mortality. These studies included 55,595 deaths among 875,561 participants. Combining data from these studies indicated no significant association between dietary cholesterol intake and CVD mortality (Pooled RR: 1.09, 95% CI: 0.96–1.24, *I*^2^ = 64.1%, *P*_heterogeneity_ = 0.004) (Table 2; Supplementary Figure 7).

In the dose–response meta-analysis based on 14 studies (nine publications) (1, 8, 15, 18, 22, 24, 50, 67, 70), we found no association between a 100 mg/day increase in cholesterol intake and CVD mortality (Pooled RR: 1.04, 95% CI: 0.99–1.10, *I*^2^ = 85.9%; *P*_heterogeneity_ < 0.001) (Table 2; Supplementary Figure 8). There was statistical evidence of a non-linear association (*P*_non-*linearity*_ = 0.009) (Figure 2; Supplementary Table 7). Accordingly, the risk of CVD mortality began to increase from 400 mg/day dietary cholesterol to higher amounts. However, the risk among these amounts was not statistically significant.

### Findings From the Meta-Analysis on Cancer Mortality

#### Egg Consumption and Cancer Mortality

The association between high vs. low egg intake and cancer mortality was examined in 24 studies (18 publications) (9, 12, 15, 19, 21, 25, 26, 48, 52, 59, 61–63, 65, 66, 69, 77, 78) with a total of 1,705,280 participants and 65,261 cancer deaths. In these studies, the median egg intake classified as “high” was 0.81 egg/day (IQR: 0.64–1.00) and 0.04 egg/day (IQR: 0–0.14) as “low,” with one study not providing quantification (59). After combining the results of these studies, a significant positive association was observed (Pooled RR: 1.23, 95% CI: 1.05–1.45, *I*^2^ = 95%, *P*_heterogeneity_ < 0.001) (Table 3; Supplementary Figure 9).

**Table 3:** Summary risk estimates for the associations between egg and cholesterol intake and risk of cancer mortality in adults aged ≥18 years^*a*^.

	**#RR^**b**^**	**Pooled RR (95% CI)^**c**^**	**I^**2**^ (%)^**d**^**	***P*-heterogeneity^**e**^**	***P*-meta-regression**
**The highest vs. lowest comparison**					
Egg intake					
Overall	15	1.23 (1.05–1.45)	95	<0.001	
Subgroup analysis					
Study location					
US	5	1.14 (1.05–1.23)	46.8	0.11	0.01
Non-US	9	1.31 (0.94–1.84)	96.4	<0.001	
Gender					
Both	11	1.25 (1.00–1.58)	96.5	<0.001	0.39
Male	2	1.09 (0.91–1.31)	22.4	0.25	
Female	2	2.17 (0.50–9.50)	89.7	0.002	
Follow-up duration					
≥15 years	8	1.14 (1.06–1.23)	51.3	0.04	0.06
<15 years	7	1.28 (0.84–1.95)	97	<0.001	
Dietary assessment tools					
FFQ or DHQ	15	1.24 (1.05–1.46)	95.3	<0.001	–
Food recall or record	0	–	–		
Adjustment for energy					
Yes	8	1.06 (0.98–1.15)	71.1	0.001	0.01
No	7	1.55 (1.08–2.22)	94.1	<0.001	
Adjustment for BMI					
Yes	11	1.23 (1.01–1.51)	96.9	<0.001	0.67
No	4	1.33 (0.95–1.87)	51.9	0.10	
Adjustment for lipid-lowering medication					
Yes	2	1.89 (0.32–1.22)	0.92.6	<0.001	0.84
No	13	1.24 (1.04–1.47)	95.7	<0.001	
Dietary cholesterol intake					
Overall	3	1.13 (1.01–1.25)	69.7	0.04	
**Linear dose-response association**					
Egg intake (per 1 egg/d increase)					
Overall	17	1.13 (1.06–1.20)	54.2	0.004	
Subgroup analysis					
Study location					
US	5	1.14 (1.07–1.21)	45.7	0.11	0.89
Non-US	11	1.11 (0.98–1.26)	62.5	0.003	
Others	1	1.20 (0.99–1.46)	–	–	
Gender					
Both	12	1.11 (1.02–1.20)	47	0.03	0.85
Male	2	1.09 (0.87–1.37)	44.4	0.18	
Female	3	1.22 (0.81–1.85)	83.5	0.002	
Follow-up duration					
≥15 years	9	1.14 (1.06–1.22)	58	0.01	0.48
<15 years	8	1.10 (0.92–1.32)	55.6	0.02	
Dietary assessment tools					
FFQ or DHQ	17	1.13 (1.06–1.20)	54.2	0.004	–
Food recall or record	0	–	–	–	
Adjustment for energy					
Yes	7	1.12 (1.06–1.17)	33.5	0.17	0.38
No	10	1.20 (1.00–1.42)	63.9	0.003	
Adjustment for BMI					
Yes	13	1.12 (1.05–1.20)	56.5	0.006	0.64
No	4	1.24 (0.92–1.66)	59	0.06	
Adjustment for lipid-lowering medication					
Yes	2	1.19 (0.51–2.71)	91.8	<0.001	0.46
No	15	1.12 (1.06–1.18)	32.3	0.11	
Dietary cholesterol intake (per 100 mg/d increase)					
Overall	3	1.06 (1.05–1.07)	0	0.95	

a *BMI, body mass index; CI, confidence interval; RR, relative risk; DHQ, dietary history questionnaire; FFQ, food frequency questionnaire; US, United States*.

b *Number of risk estimates*.

c *Obtained from the random-effects model*.

d *Inconsistency, the percentage of variation across studies due to heterogeneity*.

e *Obtained from the Q-test*.

A total of 25 cohort studies (18 publications) (9, 12, 15, 19, 21, 25, 26, 48, 52, 61–63, 65, 66, 69, 75, 76, 78) were included in the dose–response analysis. The summary RR of CVD mortality based on an increase of 1 egg/day was 1.13 (95% CI: 1.06–1.20, *I*^2^ = 54.2%, *P*_heterogeneity_ = 0.004) (Table 3; Supplementary Figure 10). There was no evidence of departure from linearity (*P*_non-*linearity*_ = 0.51) (Figure 2; Supplementary Table 6).

#### Dietary Cholesterol Intake and Cancer Mortality

A total of four prospective studies (three publications) (15, 24, 51), including 800,622 participants and 53,540 cases of cancer mortality, were identified and included in the analysis of the high (median: 330, IQR 308–480) vs. low (median: 118, IQR: 109–210) intake of dietary cholesterol and cancer mortality. Combining data from these studies indicated a positive association between cholesterol intake and cancer mortality (pooled RR: 1.13, 95% CI: 1.01–1.25, *I*^2^ = 69.7%, *P*_heterogeneity_ = 0.04) (Table 3; Supplementary Figure 11).

In the dose–response meta-analysis of the four studies, we found that a 100 mg/day increase in cholesterol intake was associated with a 6% higher risk of cancer mortality (Pooled RR: 1.06, 95% CI: 1.05–1.07, I^2^ = 0%, *P*_heterogeneity_ = 0.95) (Table 3; Supplementary Figure 12). There was no evidence of a non-linear association (*P*_non-*linearity*_ = 0.28) (Figure 2; Supplementary Table 7).

### Subgroup Analyses, Meta-Regression, Sensitivity Analyses, and Publication Bias

Tables 1–3 show findings from different subgroup and meta-regression analyses. A significant positive association was observed between egg consumption and the risk of all-cause and CVD mortality among women, and in studies conducted in the United Studies. For cancer mortality, a significant positive association was seen in studies conducted in the United States, those with a follow-up duration of ≥15 years, those that applied FFQ for dietary assessment, and studies that did not control for energy intake and lipid-lowering medication. According to the meta-regression, study location and dietary assessment methods for all-cause mortality, energy adjustment for CVD mortality, study location, follow-up duration, and energy adjustment for cancer mortality appeared to be the main sources of heterogeneity.

For cholesterol intake and all-cause CVD and cancer mortality, a significant positive association was seen in studies conducted in the United States, those with a follow-up duration of ≥15 years, and those that used FFQ for dietary assessment. Such a significant positive association was also seen for all-cause mortality among studies that controlled their analysis for energy intake and for CVD mortality among studies that did not control this variable. Based on the meta-regression, study location appeared to be the main factor responsible for the heterogeneity observed in the association between dietary cholesterol intake and all-cause mortality.

In the sensitivity analyses that excluded one study at a time from the meta-analysis, the pooled RRs were not substantially altered. Assessment of publication bias using Egger's linear regression test found no evidence for small-study effects, except for cholesterol intake and all-cause mortality. There, the application of the trim-and-fill method did not change the average effect size [(1.07, 0.95% CI: 1.02–1.13) vs. (1.07, 0.95% CI: 1.02–1.13)].

## Discussion

This large-scale systematic review and dose–response analyses included information from up to 2,772,486 participants in 55 studies. Our comprehensive analyses provide some evidence for an adverse association between high dietary intake of eggs and cholesterol with respect to all-cause and cancer mortality. However, for all outcomes, daily intakes below 0.5 eggs or 250 mg cholesterol were not associated with mortality risk, supporting the role of modest egg and cholesterol intakes as part of a healthy diet. Despite known associations between high serum cholesterol and CVD risk, we saw no evidence for any association with CVD risk, and further work is required to examine the role of cholesterol-lowering medication or functional foods, in moderating the possible effects of high dietary cholesterol intakes.

Eggs are one of the major dietary sources of cholesterol. It has been shown that dietary cholesterol can increase the plasma levels of total cholesterol and also increase the ratio of total cholesterol to HDL cholesterol (81). Since elevated plasma concentrations of cholesterol have an adverse effect on health outcomes and longevity, dietary cholesterol might have a similar effect. However, findings from epidemiological studies on the link between dietary cholesterol and longevity are conflicting. In this study, we found that egg and dietary cholesterol intake were positively associated with the risk of all-cause mortality. Two previously published meta-analyses on the relationship between egg consumption and all-cause mortality showed no evidence for an association (13, 16). This inconsistency might be explained by including a larger number of studies with a much larger number of deaths and participants in the current meta-analysis compared with previous ones. In addition, unlike the previous meta-analyses, we conducted the dose–response analyses that take into consideration the different ranges of egg and cholesterol intakes among different studies.

In the overall analyses conducted in the current meta-analysis, there was evidence of moderate-to-high between-study heterogeneity. Although the meta-regression revealed that study location and dietary assessment tools were the main sources of heterogeneity, other sources of heterogeneity might be involved. Different adjustments for key confounders (i.e., energy intake and BMI) and different follow-up durations are other sources based on the subgroup analyses. In this study, we tried to control the effects of heterogeneity on our findings by using a random-effects model for combining the risk estimates.

In fact, we found that the associations of egg and dietary cholesterol intake with all-cause mortality were non-linear. Intakes of ≤1.5 eggs/day and ≤450 mg/day dietary cholesterol did not influence the risk of all-cause mortality, while the higher amounts were associated with an increased risk of all-cause mortality. In addition to cholesterol, eggs are a rich source of nutrients, including multivitamins, minerals, and high-quality protein. However, according to our study, it seems other nutrients in the egg could not fully diminish the deleterious effects of the egg cholesterol.

Randomized controlled trials have shown that eggs and dietary cholesterol have a modest contribution to plasma concentrations of LDL cholesterol (82). Dietary cholesterol may prompt the oxidation of LDL, endothelial dysfunction, and arterial inflammation, which can raise the risk of CVD. We found no significant association between egg and dietary cholesterol intake and CVD mortality in the overall analysis. It is possible that the lack of association with CVD mortality may at least in part be explained by reverse causation bias. For example, participants might have changed their egg consumption after developing chronic diseases or other conditions related to CVD risk. There are also many cholesterol-lowering margarines containing plant sterols that may be more commonly consumed by people who are at an increased CVD risk. For the primary prevention of cardiovascular disease, major guidelines (83) recommend statins for individuals who are destined to develop atherosclerotic CVD. Increasing evidence suggests that statins with lipid- and non-lipid-related functions have cardio-protective effects (84). A few studies controlled for cholesterol-lowering medications in their analysis. Thus, the use of lipid-lowering drugs, such as statins, as a confounder could attenuate a positive association between dietary intake of eggs and cholesterol and CVD mortality.

We found that egg consumption was associated with an increased risk of cancer mortality, with a similar finding for dietary cholesterol. This is in line with previous meta-analyses on cancer risk. For example, a meta-analysis by Ruohuang et al. reported that egg consumption was associated with an increased risk of breast cancer among the European, Asian, and postmenopausal populations (85). There were two other reports indicating a positive link between egg consumption and cancer risk (86, 87). In terms of dietary cholesterol, in a meta-analysis of cohort studies, Wang et al. showed that the intake of red meat and processed meat, known as rich sources of cholesterol, was positively associated with the risk of cancer mortality (88). Preclinical studies have suggested that deregulation of cholesterol homeostasis is an important contributing factor to cancer development and mortality (89). Based on these studies, increased cellular cholesterol levels, which may result from high dietary cholesterol intake or disrupted cholesterol homeostasis, can lead to increased cancer cell proliferation (90, 91).

Despite evidence for a positive association between egg and dietary cholesterol intake and cancer risk or mortality, there was notable heterogeneity and the significant association was not observed in several studies (19, 24, 51, 59, 61). These inconsistencies may be explained by different characteristics of included studies, such as different follow-up periods and different adjustments in statistical analysis. For instance, in the subgroup analyses, we found a significant positive association between egg consumption and cancer mortality in studies with a follow-up duration of ≥15 years but not in those that had a short follow-up period (<15 years). Further high-quality studies are needed to confirm our findings.

## Strengths and Limitations

We conducted a comprehensive meta-analysis of prospective studies, which included more than 2.7 million participants and 228,425 deaths. With this comprehensive data, we were able to conduct linear and non-linear dose–response analyses, which allowed us to determine dose–response effects and the shape of the possible associations. However, some limitations should be considered when interpreting the current results. First, since our included studies were observational in nature, causality cannot be established. Second, the role of residual confounders resulting from unmeasured behavioral and biological factors or errors in the measurement of covariates cannot be entirely excluded owing to the observational design of included studies. Third, although most studies had controlled for potential confounders, adjustment strategies were not consistent across the studies, and some did not consider dietary variables typically associated with egg intake (e.g., intakes of processed and unprocessed meats), which would have affected their findings. Fifth, we did not have data on cooking methods for eggs or feeding methods for chicken that could alter the nutritional composition of the eggs. Sixth, findings might not be generalizable to low- or middle-income economics, in which diets are carbohydrate-rich, and consumption of protein sources is low. Finally, the majority of studies used a questionnaire to estimate the intake of eggs.

## Conclusion

These data suggest possible increases in all-cause and cancer mortality with high daily intake of eggs and cholesterol. However, intakes of ≤1.5 eggs/day and ≤450 mg/day of dietary cholesterol did not influence the risk of all-cause/CVD mortality. Due to small risk estimates and moderate between-study heterogeneity in some associations, our findings on all-cause mortality should be considered with caution. Further studies are needed to confirm our findings regarding CVD mortality.

## Author's Note

Findings from previous studies on egg consumption and the risk of mortality have been inconclusive. A high intake of eggs and dietary cholesterol is associated with an increased risk of mortality from all causes and cancer. Egg consumption was not associated with cardiovascular disease risk. Each additional egg per day was associated with a 5% higher risk of all-cause and a 13% higher risk of cancer mortality. Each 100 mg/day increment in dietary cholesterol intake was associated with a 6% higher risk of all-cause mortality and a 6% higher risk of cancer mortality.

## Data Availability Statement

The original contributions presented in the study are included in the article/Supplementary Material, further inquiries can be directed to the corresponding author/s.

## Author Contributions

MDM and KL contributed to the literature search and data extraction. SN and OS contributed to data analysis. OS and SN drafted the manuscript, which was critically revised for important intellectual content by all authors. EH and JB contributed to the methodology, statistical analysis, and manuscript drafting. OS supervised the study. SN is a guarantor and had full access to all the data and takes responsibility for the integrity of the data and the accuracy of the data analysis. All authors have read and approved the final manuscript.

## Funding

The meta-analysis was funded by the Gerash University of Medical Sciences, Gerash, Iran. The funder had no role in the design and conduct of the study; collection, management, analysis, and interpretation of the data; preparation, review, or approval of the manuscript; or the decision to submit the manuscript for publication.

## Conflict of Interest

The authors declare that the research was conducted in the absence of any commercial or financial relationships that could be construed as a potential conflict of interest.

## Publisher's Note

All claims expressed in this article are solely those of the authors and do not necessarily represent those of their affiliated organizations, or those of the publisher, the editors and the reviewers. Any product that may be evaluated in this article, or claim that may be made by its manufacturer, is not guaranteed or endorsed by the publisher.
