# Supplementary material for: Egg and Dietary Cholesterol Intake and Risk of All-Cause, Cardiovascular, and Cancer Mortality: A Systematic Review and Dose-Response Meta-Analysis of Prospective Cohort Studies
Source: Front Nutr. 2022 May 27;9:878979. doi: 10.3389/fnut.2022.878979 (PMC9195585; doi:10.3389/fnut.2022.878979)
Supplement: Supplementary file 1 [file Data_Sheet_1.docx]

**Supplemental Table 1**: Medical subject headings (MeSH) and non-MeSH terms used to search relevant publications on the relation between egg and cholesterol intake and risk of mortality^1^

| Database | Step | Terms | Results |
| --- | --- | --- | --- |
| PubMed | 1 | ("Egg"[All fields] OR "Eggs"[All fields] OR "dietary cholesterol"[All fields] OR "cholesterol consumption"[All fields] OR "cholesterol intake"[All fields] OR “protein sources"[All fields]) |  |
|  | 2 | ("Mortality"[tiab] OR "Death"[tiab] OR "fatal"[tiab] OR "Survival"[tiab] OR "lethal"[tiab] OR "Mortality"[Subheading] OR "Neoplasms"[tiab] OR "cancer Survivors"[tiab] OR "cardiovascular diseases"[tiab] OR "coronary disease"[tiab] OR "Myocardial Ischemia"[tiab] OR "coronary artery disease"[tiab] OR "myocardial infarction"[tiab] OR "stroke"[tiab] OR "mortality"[Mesh] OR "death"[Mesh] OR "cardiovascular diseases"[Mesh] OR "coronary disease"[Mesh] OR "Myocardial Ischemia"[Mesh] OR "coronary artery disease"[Mesh] OR "myocardial infarction"[Mesh] OR "stroke"[Mesh]) |  |
|  | 3 | ("cohort"[All fields] OR "cohorts"[All fields] OR "prospective"[All fields] OR "longitudinal"[All fields] OR "follow-up"[All fields] OR "population-based"[All fields] OR "relative risk"[All fields] OR "odds ratio"[All fields] OR "hazard ratio"[All fields] OR "incidence rate ratio"[All fields]) |  |
|  | 4 | #1 AND #2 AND #3 | 1318 |
| Scopus | 1 | (TITLE-ABS-KEY(egg) OR TITLE-ABS-KEY(eggs) OR TITLE-ABS-KEY("cholesterol consumption") OR TITLE-ABS-KEY("dietary cholesterol") OR TITLE-ABS-KEY("cholesterol intake") OR TITLE-ABS-KEY("protein sources") |  |
|  | 2 | TITLE-ABS-KEY(Mortality) OR TITLE-ABS-KEY(Death) OR TITLE-ABS-KEY(fatal) OR TITLE-ABS-KEY(Survival) OR TITLE-ABS-KEY(lethal) OR TITLE-ABS-KEY("cardiovascular diseases") TITLE-ABS-KEY ("Neoplasms") OR TITLE-ABS-KEY ("cancer Survivors") OR TITLE-ABS-KEY("coronary disease") OR TITLE-ABS-KEY("Myocardial Ischemia") OR TITLE-ABS-KEY("coronary artery disease") OR TITLE-ABS-KEY("myocardial infarction") OR TITLE-ABS-KEY("stroke") |  |
|  | 3 | TITLE-ABS-KEY("case-control") OR TITLE-ABS-KEY(cohort) OR TITLE-ABS-KEY(cohorts) OR TITLE-ABS-KEY(prospective) OR TITLE-ABS-KEY(longitudinal) OR TITLE-ABS-KEY(retrospective) OR TITLE-ABS-KEY("follow-up") OR TITLE-ABS-KEY("cross-sectional") OR TITLE-ABS-KEY("population-based") OR TITLE-ABS-KEY("relative risk") OR TITLE-ABS-KEY("odds ratio") OR TITLE-ABS-KEY("hazard ratio") OR TITLE-ABS-KEY("incidence rate ratio")) |  |
|  | 4 | #1 AND #2 AND #3 | 2129 |
| Web of Science | 1 | TS=(egg OR eggs OR "dietary cholesterol" OR "cholesterol intake" OR "cholesterol consumption" OR "protein sources") |  |
|  | 2 | TS=(mortality OR death OR fatal OR survival OR "neoplasms" OR "cancer survivors" OR "cardiovascular diseases" OR "coronary disease" OR "Myocardial Ischemia" OR "coronary artery disease" OR "myocardial infarction" OR stroke) |  |
|  | 3 | TS=(cohort OR cohorts OR "case-control" OR prospective OR longitudinal OR retrospective OR "follow-up" OR "population-based" OR "cross-sectional" OR "relative risk" OR "odds ratio" OR "hazard ratio" OR "incidence rate ratio") |  |
|  | 4 | #1 AND #2 AND #3 | 1822 |
| Google scholar |  | Egg and mortality | 500 |
|  |  | Cholesterol intake and mortality | 500 |
|  |  | By searching the above combination in this engine, we screened the first 500 relevancy ranked papers to avoid missing any eligible studies. |  |
| Total |  |  | 6269 |

^1^ Two investigators (MDM & SN) searched the online databases independently.

**Supplemental table 2**: Characteristics of included studies on the associations between egg and cholesterol intake and all-cause mortality in adults aged >18 years

| Author | Country | Age* | Sample size | Follow up (y) ^¥^ | Deaths | Exposure | Exposure assessment | Median/cutoff point | RR (95%CI) | Adjustment^†^ |
| --- | --- | --- | --- | --- | --- | --- | --- | --- | --- | --- |
| Dehghan et al. 2020 | 21 countries | 50.6 | M/F: 146011 | 9.5 | 8932 | Egg | FFQ | <1 n/wk  1–<3 n/wk  3–<5 n/wk  5–<7 n/wk  ≥7 n/wk | 1  0.95 (0.89-1.02)  0.94 (0.87-1.02)  0.99 (0.90-1.09)  0.99 (0.91-1.09) | Age, gender, smoking, location, education, physical activity, history of diabetes, daily intakes of fruits, vegetables, dairy, red meat, poultry, and fish, percentage energy from carbohydrate, total daily energy, and center |
|  |  |  |  |  |  | Dietary cholesterol |  | <100 mg/d  100-200 mg/d  200-300 mg/d  300-400 mg/d  >400 mg/d | 1  0.96 (0.86-1.06)  0.94 (0.83-1.06)  1.00 (0.87-1.14)  1.05 (0.91-1.22) |  |
| Nakamura et al. 2017 | Japan | ≥30 | F:  4686 | 15 | 599 | Egg | Food record | <1/wk  1–2/wk  1/2d  1/d  ≥2/d | 0.98 (0.70-1.37)  0.90 (0.73-1.10)  0.96 (0.78-1.19)  1  2.05 (1.20-3.52) | Age, BMI, hypertension, diabetes, cigarette smoking, alcohol drinking, dyslipidemia therapy, intake of fiber, meat, and sodium |
| Nakamura et al.2004 | Japan | ≥30 | M/F: 9263 | 14 | 1202 | Egg | FFQ | Men: ≥2/d  1/d  1/2d  1-2/wk  seldom | 0.89 (0.57-1.38)  1  0.89 (0.72-1.08)  0.94 (0.78-1.13)  0.73 (0.48-1.12) | Age, serum creatinine, total cholesterol, blood glucose, BMI, SBP, DBP, use of blood pressure-lowering drugs, cigarette smoking, and alcohol intake |
|  |  |  |  |  |  |  |  | Women: ≥2/d  1/d  1/2d  1-2/wk  seldom | 1.48 (0.84-2.61)  1  1.00 (0.81-1.24)  0.78 (0.63-0.96)  0.97 (0.72-1.32) |  |
| Xu et al.  2018 | China | ≥50 | M/F: 18707 | 9.8 | 2685 | Egg | FFQ | <1 n/wk  1–2 n/wk  3–4 n/wk  5–6 n/wk  ≥7 n/wk | 1  1.01 (0.90-1.14)  0.98 (0.85-1.12)  0.94 (0.76-1.17)  1.11 (0.93-1.33) | Gender, age, education, occupation, family income, smoking status, physical activity, alcohol drinking, self-rated health and chronic disease history (diabetes, hypertension and dyslipidemia), total energy, vegetable, fruit, milk and nut intake |
| Shi et al. 2015 | China | >80 | M/F: 8959 | 13 | 6626 | Egg | FFQ | Never  Occasionally  Daily | 1  1.07 (0.99-1.14)  1.02 (0.94-1.10) | Age, gender, job before 60 years of age, residence, smoking, alcohol drinking, physical activity (regular exercise), number of chronic diseases, frequency intake of fruit, vegetable |
| Qureshi et al. 2006 | US | 25-74 | M/F: 9734 | 20 | 3177 | Egg | Nutritional habits questionnaire | <1 n/wk  1-6 n/wk  >6 n/wk | 1  0.90 (0.80-1.00)  1.00 (0.90-1.10) | Age, gender, race/ethnicity, SBP, diabetes mellitus, serum cholesterol, cigarette smoking, BMI, and educational status |
| Mann et al. 1997 | UK | 16-79 | M/F: 10802 | 13.3 | 392 | Egg | FFQ | <1 n/wk  1-5 n/wk  ≥6 n/wk | 1  0.74 (0.58-0.96)  0.92 (0.68–1.23) | Age, gender, smoking, social class |
|  |  |  |  |  |  | Dietary cholesterol |  | 155.6 mg/d  277.1 mg/d  431.4 mg/d | 1  0.74 (0.56–0.97)  1.02 (0.78–1.34) |  |
| Zamora‑Ros et al. 2018 | Spain | 29-69 | M/F: 40621 | 18.4 | 3561 | Egg | Diet history | <12.6 g/d  12.6-23.35 g/d  23.35-36.5 g/d  >36.5 g/d | 1  1.08 (0.99-1.18)  1.02 (0.93-1.12)  1.01 (0.91-1.11) | Center, age at recruitment in 5 year categories, gender, smoking intensity, BMI, lifetime alcohol intake, education level, physical activity, energy intake, and adherence to Mediterranean diet |
| Sun et al. 2021 | US | 50-79 | F:  102521 | 18.1 | 25976 | Egg | FFQ | 0.03 oz./d  0.1 oz./d  0.2 oz./d  0.3 oz./d  0.7 oz./d | 1  0.99 (0.96-1.03)  1.01 (0.97-1.06)  1.02 (0.98-1.06) 1.13 (1.08-1.18) | Age at baseline, race/ethnicity, education, income, Observational Study/Clinical Trials, unopposed estrogen use, estrogen+progesterone use, smoking status, physical activity, alcohol intake, total energy intake, baseline diabetes mellitus status, baseline high blood cholesterol status, family history of heart attack/stroke, whole grain consumption, vegetable consumption, fruit consumption, sugar-sweetened beverage consumption, mutual adjustment for other protein sources, body mass index |
| Ruggiero et al. 2021 | Italy | ≥ 35 | M/F: 20562 | 8.2 | 838 | Egg | FFQ | >0≤1 n/wk  >1≤2 n/wk  >2≤4 n/wk  >4 n/wk | 1  1.14 (0.96–1.36)  1.22 (1.01–1.46)  1.50 (1.13–1.99) | Age, gender, energy intake, educational level, household income, residence, smoking, BMI, leisure-time PA, baseline diabetes, hypertension, hyperlipidemia, and Mediterranean diet score |
| Zhong et al. 2019 | 6 cohorts | 51.6 | M/F: 29615 | 17.5 | 6132 | Egg | FFQ | 0 n/d  0.067 n/d  0.143 n/d  0.429 n/d  1 n/d | 1  0.96 (0.88-1.05)  0.97 (0.90-1.06)  1.02 (0.94-1.10)  1.10 (1.01-1.19) | Age, gender, race/ethnicity, education, total energy, smoking status, smoking pack-years, cohort-specific physical activity z-score, alcohol consumption, use of hormone replacement therapy, BMI, diabetes status , SBP, use of anti-hypertensive medications, high density lipoprotein cholesterol (HDL-C), non-HDL-C, and use of lipid-lowering medications |
|  |  |  |  |  |  | Dietary cholesterol |  | 114 mg/d  174 mg/d  229 mg/d  300 mg/d  453 mg/d | 1  1.02 (0.94-1.11)  1.00 (0.92-1.09)  1.03 (0.94-1.13)  1.18 (1.07-1.30) |  |
| Van den Brandt et al. 2019 | Netherlands | 55-69 | M/F: 12025 | 10 | 8823 | Egg | FFQ | 0 g/d  7.1 g/d  14.2 g/d  21.4 g/d | 1  0.78 (0.64-0.96)  0.83 (0.68-1.01)  0.87 (0.71–1.06) | Age at baseline, gender, cigarette smoking status, history of physician-diagnosed hypertension and diabetes, body height, BMI, non-occupational physical activity, highest level of education, intake of alcohol, vegetables and fruit, energy, use of nutritional supplements, and postmenopausal HRT |
| Xia et al. 2020 | China | 51.5 | M/F: 102136 | 7.6 | 5511 | Egg | FFQ | <1 n/wk  1–<3 n/wk  3–<6 n/wk  6–<10 n/wk  ≥10 n/wk | 1.29 (1.18-1.41)  1.07 (0.99-1.16)  1  1.13 (1.04-1.24)  1.13 (1.04-1.24) | Age, gender, urban or rural resident, per capita household income, education attainment, tobacco smoking alcohol consumption and family history of CVD, physical activity, BMI and dietary factors (red meat intake, fresh fruit and vegetable intake) |
| Xia et al. 2020 | US | 50.2 | M/F: 37121 | 7.8 | 4991 | Egg | Food recall | <0.5 n/d  ≥0.5 to <1 n/d  ≥1 n/d | 1  0.96 (0.88-1.05)  1.05 (0.90-1.23) | Age, gender, race/ethnicity, education, family income poverty ratio, marital status, NHANES cycles, total energy intake, cigarette smoking, alcohol drinking, physical activity, BMI, hypertension, diabetes, hypercholesterolemia, CVD, and cancer |
|  |  |  |  |  |  | Dietary cholesterol |  | 183.5 mg/d  235.4 mg/d  277.3 mg/d  325.5 mg/d  419.5 mg/d | 1  0.89 (0.80-0.98) 0.90 (0.80-1.01)  1.04 (0.92-1.18)  1.07 (0.89-1.28) |  |
| Zhuang et al. 2021 | US | 62.2 | M/F: 521120 | 16 | 129328 | Egg | FFQ | 0 g/2000 kcal/d  3 g/2000 kcal/d  7.5 g/2000 kcal/d  13.7 g/2000 kcal/d  28.7 g/2000 kcal/d | 1  1.01 (1.00-1.03)  1.05 (1.03-1.07)  1.07 (1.05-1.09)  1.14 (1.12-1.16) | Age, gender, BMI, race, education, marital, household income, smoking, alcohol, vigorous physical activity, usual activity at work, and history of hypertension, high cholesterol level, heart disease, stroke, diabetes, cancer at baseline, total energy, egg whites/substitutes, red meat, fish, poultry, and dairy products, fruit, vegetables, potatoes, nuts/legumes, whole grains, refine grains, coffee, and sugar-sweetened beverages |
|  |  |  |  |  |  | Dietary cholesterol |  | 118.3 mg/2000 kcal  168.7 mg/2000 kcal/d  207.9 mg/2000 kcal/d  252 mg/2000 kcal/d  330 mg/2000 kcal/d | 1  1.01 (0.99-1.03)  1.03 (1.01-1.05)  1.06 (1.04-1.09)  1.14 (1.11-1.17) | Age, gender, BMI, race, education, marital, household income, smoking, alcohol, vigorous physical activity, usual activity at work, and history of hypertension, high cholesterol level, heart disease, stroke, diabetes, cancer at baseline, total energy and intakes of saturated fat, polyunsaturated fat, monounsaturated fat, trans fat, animal protein, fiber, and sodium |
| Zhuang et al. 2019 | China | >20 | M/F: 18914 | 15 | 1429 | Egg | Food record | ≤4.8 g./2000 kcal^.^d  4.9-20.7 g./2000 kcal^.^d  20.8-36.3 g./2000 kcal^.^d  36.4-60.9 g./2000 kcal^.^d  ≥61 g./2000 kcal^.^d | 1  0.66 (0.56-0.77)  0.64 (0.54-0.75)  0.69 (0.58-0.83)  0.69 (0.59-0.81) | Age and gender, nationality, marital status, BMI, household income, urbanization index, education, physical activity, smoking, alcohol drinking status, history of CVD, cancer, diabetes, and hypertension ,total energy, intakes of protein, saturated fat, polyunsaturated fat, monounsaturated fat, sodium and fiber. |
|  |  |  |  |  |  | Dietary cholesterol |  | ≤0.13 g./2000 kcal^.^d  0.13-0.23 g./2000 kcal^.^d  0.23-0.33 g./2000 kcal^.^d  0.33-0.48 g./2000 kcal^.^d  ≥0.48 g./2000 kcal^.^d | 1  0.84 (0.72-0.99)  0.84 (0.70-1.00)  1.02 (0.85-1.22)  0.89 (0.73-1.08) |  |
| Guo et al. 2017 | UK | 45-59 | M:  1781 | 22.8 | 1028 | Egg | FFQ | 0 ≤ n ≤ 1/wk  1 < n ≤ 2/wk  2 < n ≤ 3/wk  3 < n < 5/wk  n ≥ 5/wk | 1  1.08 (0.87, 1.34)  1.20 (0.98, 1.49)  1.02 (0.81, 1.29)  1.08 (0.84, 1.38) | Age, BMI, total energy intake, alcohol consumption, smoking, energy expenditure, social class, family history of myocardial infarction, diabetes mellitus, sugar intake, fruit consumption, red meat consumption and fiber (cereal and vegetable sources) |
| Kahn et al. 1984 | US | >30 | M/F: 22033 | 21 | 6075 | Egg | Frequency questionnaire | <1 days/wk  1-2 days/wk  3-5 days/wk  6-7 days/wk | 1  0.94 (NR)  0.96 (NR)  1.18 (1.00-1.38) | Age, gender, history of heart disease, stroke, hypertension, diabetes, cancer, age at initial exposure, smoking history |
| Farvid et al. 2017 | Iran | 36-85 | M/F: 42403 | 11 | 3291 | Egg | FFQ | 0 serving /d  0.06 serving/d  0.18 serving /d  0.48 serving /d | 1  0.92 (0.84-1.02)  0.90 (0.82-0.99)  0.88 (0.79-0.97) | Age, gender, ethnicity, education, marital status, residency, smoking, opium use, alcohol, BMI, SBP, occupational physical activity, family history of cancer, wealth score, medication, energy intake |
| Djousse et al. 2008 | US | 53.7 | M: 21327 | 20 | 5169 | Egg | FFQ | <1 serving /wk  1 serving /wk  2-4 serving /wk  5-6 serving /wk  ≥7 serving /wk | 1  0.94 (0.87-1.02)  1.03 (0.95-1.11)  1.05 (0.93-1.19)  1.23 (1.11-1.36) | Age, BMI, smoking, history of hypertension, vitamin intake, alcohol consumption, vegetable consumption, breakfast cereal, physical activity, treatment arm, atrial fibrillation, diabetes mellitus, hypercholesterolemia, parental history of premature myocardial infarction |
| Chen et al. 2020 | US | 50–79 | F: 96831 | 18.9 | 19508 | Dietary cholesterol | FFQ | 73.1 mg/1000 kcal/d  100 mg/1000 kcal/d  120.9 mg/1000 kcal/d  145.4 mg/1000 kcal/d  193.2 mg/1000 kcal/d | 1  0.99 (0.94-1.03)  1.00 (0.95-1.05)  1.05 (1.00-1.11) 1.09 (1.02-1.15) | Age, region, race/ethnicity, study group, education, annual family income, health insurance, smoking status, pack-years of smoking, alcohol consumption, quitting smoking/drinking owing to health problems, recreational physical activity, total energy intake, using fat to deep fry/pan fry/sauté, aspirin use, use of nonsteroidal anti-inflammatory drugs, hormone use, self-rated health status, waist circumference, diabetes, systolic and diastolic blood pressure, antihypertensive drug use, dyslipidemia, energy-adjusted fiber, saturated fat, polyunsaturated fat, monounsaturated fat, trans fat, animal protein, sodium) |
| Fortes et al. 2000 | Italy | >65 | M/F: 162 | 5 | 53 | Dietary cholesterol | FFQ | NR | 1  1.24 (0.62-2.48)  0.89 (0.39-2.03) | - |
| Knoops et al. 2006 | Europe | 70-90 | M/F: 3117 | 10 | 1382 | Dietary cholesterol | Diet history | ≥269 mg/d  <269 mg/d | 0.99 (0.91-1.07)  1 | Age, gender, physical activity, smoking, alcohol use, number of years of education, BMI, chronic disease at baseline and study center |
| Virtanen et al. 2019 | Finland | 42-60 | M:2641 | 22.3 | 1225 | Egg | Food record | 8 g/d  20 g/d  34 g/d  59 g/d | 1  0.85 (NR)  0.83 (NR)  0.89 (0.75-1.05) | Age, examination year, energy intake, income, education years, marital status, leisure-time physical activity, pack-years of smoking, alcohol intake, BMI, diagnosis of type 2 diabetes, cardiovascular disease, cancer, or hypertension or use of cardiac, hypercholesterolemia, hypertension, or diabetes medications, intakes of fiber and saturated, monounsaturated, polyunsaturated, and trans fatty acids |
| Zupo et al. 2020 | Italy | 48 | M/F:  2472 | 34 | 990 | Egg | FFQ | Per 1 egg/day | 0.86 (0.76-0.99) | Gender, (Female), Age, BMI, Education, Smoking, Comorbidity, Wine, and Olive oil |
| Wang et al. 2016 | China | 40-69 | M/F:  2445 | 26 | 1501 | Egg | FFQ | 4 times/month | 1.01 (0.98-1.03) | Age, gender, commune, smoking, drinking, season and body mass index |
| Trichopoulou et al. 2006 | Europa | NR | M/F:  1013 | 4.5 | 80 | Egg | FFQ | Per 10 g/d | 1.31 (1.07-1.60) | Gender, age, educational level, smoking, waist-to-height, hip circumference, physical activity, , treatment with insulin, treatment for hypertension at enrolment, and treatment for hypercholesterolemia at enrolment, all other indicated food groups including flour, flakes, starches, pasta, rice, other grain, bread, crispbread, rusks, breakfast cereals, biscuits, dough and pastry, etc. |
| Iribarren et al. 1996 | Japan | 59.9 | M:  6137 | 16 | 1490 | Dietary cholesterol | 24-hour recall | Per 300 mg/d | 1.17(1.06-1.29) | Alcohol consumption, energy intake, BMI |
| Bongard et al. 2016 | French | 55.5 | M:  960 | 14.8 | 150 | Egg | Food record | NR | 1  1.00 (0.63-1.59)  0.67 (0.40-1.12)  1.11 (0.70-1.76) | Center, age, payment of income tax, obesity, alcohol consumption, smoking habits, physical activity, presence of a serious chronic condition and diet quality score |
| Sluik et al. 2014 | Europe | 35-70 | M/F: 6,384 | 9.9 | 830 | Egg | FFQ | Per 10 g/d | 1.04 (0.96, 1.12) | Age- and centre-stratified and adjusted for gender, prevalence of heart disease, cancer or stroke, educational attainment, diabetes medication use  (in diabetic individuals), and the following when there were no exposure variables: alcohol consumption, smoking behaviour, physical activity and  underlying dietary patterns |
|  |  |  | M/F: 258,911 |  | 12135 |  |  | Per 10 g/d | 1.09 (1.06, 1.12) |  |

Abbreviation: RR: Relative Risk - CI: confidence interval- M: male- F: female- FFQ: food frequency questionnaire- BMI: body mass index- US: United States- NR: not-reported- wk: week- SBP: systolic blood pressure

*Presented as mean or rang

**Supplemental table 3**: Characteristics of included studies on the association between egg and cholesterol intake and CVD mortality in adults aged >18 years

| Author | Country | Age* | Sample size | Follow up (y) ^¥^ | Deaths | Exposure | Exposure assessment | Median/cutoff point | RR (95%CI) | Adjustment^†^ |
| --- | --- | --- | --- | --- | --- | --- | --- | --- | --- | --- |
| Dehghan et al. 2020 | 21 countries | 50.6 | M/F: 146011 | 9.5 | 3410 | Egg | FFQ | <1 n/wk  1–<3 n/wk  3–<5 n/wk  5–<7 n/wk  ≥7 n/wk | 1  0.95 (0.84, 1.08)  0.91 (0.78, 1.06)  0.92 (0.77, 1.10)  1.00 (0.85, 1.19) | Age, gender, smoking, location, education, physical activity, history of diabetes, daily intakes of fruits, vegetables, dairy, red meat, poultry, and fish, percentage energy from carbohydrate, total daily energy, and center |
|  |  |  |  |  |  | Dietary cholesterol |  | <100 mg/d  100-200 mg/d  200-300 mg/d  300-400 mg/d  >400 mg/d | 1  0.96 (0.81-1.13)  0.88 (0.72-1.08)  0.84 (0.67-1.05)  0.98 (0.77-1.23) |  |
| Nakamura et al. 2017 | Japan | ≥30 | F:  4686 | 15 | 183 | Egg | Food record | <1/wk  1–2/wk  1/2d  1/d  ≥2/d | 1.09 (0.60-1.97)  1.16 (0.81-1.67)  0.92 (0.61-1.38)  1  1.24 (0.38-4.10) | Age, BMI, hypertension, diabetes, cigarette smoking, alcohol drinking, dyslipidemia therapy, intake of fiber, meat, and sodium |
| Nakamura et al.2004 | Japan | ≥30 | M/F: 9263 | 14 | 299 | Egg | FFQ | Stroke death (Men)  ≥2/d  1/d  1/2d  1-2/wk  Seldom | 0.25 (0.03- 1.81)  1  1.10 (0.68- 1.76)  1.09 (0.69- 1.72)  0.93 (0.36- 2.40) | Age, serum creatinine, total cholesterol, blood glucose, BMI, SBP, DBP, use of blood pressure-lowering drugs, cigarette smoking, and alcohol intake |
|  |  |  |  |  |  |  |  | Stroke death (Women)  ≥2/d  1/d  1/2d  1-2/wk  Seldom | 1.22 (0.29-5.17)  1  1.46 (0.89-2.40)  0.79 (0.47- 1.33)  0.78 (0.35- 1.73) |  |
|  |  |  |  |  |  |  |  | IHD death (Men)  ≥2/d  1/d  1/2d  1-2/wk  Seldom | -  1  1.49 (0.63- 3.48)  1.71 (0.78- 3.76)  1.18 (0.26- 5.42) |  |
|  |  |  |  |  |  |  |  | IHD death (Women)  ≥2/d  1/d  1/2d  1-2/wk  Seldom | 1.27 (0.16-9.80)  1  0.78 (0.35- 1.82)  0.64 (0.28- 1.44)  1.42 (0.56- 3.62) |  |
| Xu et al.  2018 | China | ≥50 | M/F: 18707 | 9.8 | 873 | Egg | FFQ | <1 n/wk  1–2 n/wk  3–4 n/wk  5–6 n/wk  ≥7 n/wk | 1  1.14 (0.93-1.4)  1.07 (0.84-1.35)  0.96 (0.66-1.41)  1.05 (0.76-1.45) | Gender, age, education, occupation, family income, smoking status, physical activity, alcohol drinking, self-rated health and chronic disease history (diabetes, hypertension and dyslipidemia), total energy, vegetable, fruit, milk and nut intake |
| Mann et al. 1997 | UK | 16-79 | M/F: 10802 | 13.3 | 64 | Egg | FFQ | <1 n/wk  1-5 n/wk  ≥6 n/wk | 100  1.28 (0.59-2.79)  2.68 (1.19-6.02) | Age, gender, smoking, social class |
|  |  |  |  |  |  | Dietary cholesterol |  | 155.6 mg/d  277.1 mg/d  431.4 mg/d | 100  1.81 (0.77-4.29)  3.53 (1.57-7.96) |  |
| Zamora‑Ros et al. 2018 | Spain | 29-69 | M/F: 40621 | 18.4 | 761 | Egg | Diet history | <12.6 g/d  12.6-23.35 g/d  23.35-36.5 g/d  >36.5 g/d | 1  1.11 (0.91-1.34)  1.10 (0.90-1.34)  1.07 (0.86-1.32) | Center, age at recruitment in 5 year categories, gender, smoking intensity, BMI, lifetime alcohol intake, education level, physical activity, energy intake, and adherence to Mediterranean diet |
| Sun et al. 2021 | US | 50-79 | F:  102521 | 18.1 | 6993 | Egg | FFQ | 0.03 oz./d  0.1 oz./d  0.2 oz./d  0.3 oz./d  0.7 oz./d | 1  1.05 (0.96-1.12)  1.08 (0.99-1.17)  1.13 (1.03-1.20)  1.24 (1.14-1.34) | Age at baseline, race/ethnicity, education, income, Observational Study/Clinical Trials, unopposed estrogen use, estrogen+progesterone use, smoking status, physical activity, alcohol intake, total energy intake, baseline diabetes mellitus status, baseline high blood cholesterol status, family history of heart attack/stroke, whole grain consumption, vegetable consumption, fruit consumption, sugar-sweetened beverage consumption, mutual adjustment for other protein sources, body mass index |
| Ruggiero et al. 2021 | Italy | ≥ 35 | M/F: 20562 | 8.2 | 271 | Egg | FFQ | >0≤1 n/wk  >1≤2 n/wk  >2≤4 n/wk  >4 n/wk | 1  1.29 (0.94-1.77)  1.43 (1.03-1.97)  1.75 (1.07-2.87) | Age, gender, energy intake, educational level, household income, residence, smoking, BMI, leisure-time PA, baseline diabetes, hypertension, hyperlipidaemia, and Mediterranean diet score |
| Van den Brandt et al. 2019 | Netherlands | 55-69 | M/F: 6187 | 10 | 2985 | Egg | FFQ | 0 g/d  7.1 g/d  14.2 g/d  21.4 g/d | 1  0.89 (0.69-1.16)  0.90 (0.70-1.16)  0.92 (0.71-1.19) | Age at baseline, gender, cigarette smoking status, history of physician-diagnosed hypertension and diabetes, body height, BMI, non-occupational physical activity, highest level of education, intake of alcohol, vegetables and fruit, energy, use of nutritional supplements, and postmenopausal HRT |
| Xia et al. 2020 | US | 50.2 | M/F: 37121 | 7.8 | 870 | Egg | Food recall | <0.5 n/d  ≥0.5 to <1 n/d  ≥1 n/d | 1  0.97 (0.81-1.17)  0.89 (0.64-1.23) | Age gender, race/ethnicity, education, family income poverty ratio, marital status, NHANES cycles, total energy intake, cigarette smoking, alcohol drinking, physical activity, BMI, hypertension, diabetes, hypercholesterolemia, CVD, and cancer |
|  |  |  |  |  |  | Dietary cholesterol |  | 183.5 mg/d  235.4 mg/d  277.3 mg/d  325.5 mg/d  419.5 mg/d | 1  0.99 (0.77-1.28)  0.87 (0.67-1.13)  1.06 (0.77-1.48)  1.01 (0.70-1.46) |  |
| Zhuang et al. 2021 | US | 62.2 | M/F: 521120 | 16 | 38747 | Egg | FFQ | 0 g/2000 kcal/d  3 g/2000 kcal/d  7.5 g/2000 kcal/d  13.7 g/2000 kcal/d  28.7 g/2000 kcal/d | 1  1.00 (0.96-1.03)  1.00 (0.97-1.04)  1.06 (1.03-1.10)  1.15 (1.11-1.19) | Age, gender, BMI, race, education, marital, household income, smoking, alcohol, vigorous physical activity, usual activity at work, and history of hypertension, high cholesterol level, heart disease, stroke, diabetes, cancer at baseline, total energy, egg whites/substitutes, red meat, fish, poultry, and dairy products, fruit, vegetables, potatoes, nuts/legumes, whole grains, refine grains, coffee, and sugar-sweetened beverages |
|  |  |  |  |  |  | Dietary cholesterol |  | 118.3 mg/2000 kcal/d  168.7 mg/2000 kcal/d  207.9 mg/2000 kcal/d  252 mg/2000 kcal/d  330 mg/2000 kcal/d | 1  1.00 (0.97-1.04)  1.01 (0.97-1.04)  1.06 (1.02-1.10)  1.12 (1.08-1.18) | Age, gender, BMI, race, education, marital, household income, smoking, alcohol, vigorous physical activity, usual activity at work, and history of hypertension, high cholesterol level, heart disease, stroke, diabetes, cancer at baseline, total energy and intakes of saturated fat, polyunsaturated fat, monounsaturated fat, trans fat, animal protein, fiber, and sodium |
| Farvid et al. 2017 | Iran | 36-85 | M/F: 42403 | 11 | 1467 | Egg | FFQ | 0 serving /d  0.06 serving/d  0.18 serving /d  0.48 serving /d | 1   - 1. (0.87-1.16)   0.93 (0.80-1.08)  0.92 (0.79-1.07) | Age, gender, ethnicity, education, marital status, residency, smoking, opium use, alcohol, BMI, systolic blood pressure, occupational physical activity, family history of cancer, wealth score, medication, energy intake |
| Goldberg et al. 2014 | US | >40 | M/F: 2669 | 11 | 452 | Egg | FFQ | <1 n/mo  1 n/mo  2-3 n/mo  1 n/wk  ≥2 n/mo | 1  0.98 (0.74-1.28)  0.78 (0.56-1.08)  1.09 (0.84-1.41)  1.00 (0.57-1.77) | Age, gender, race/ethnicity, BMI, diabetes, hypertension, LDL, HDL, TG, cholesterol-lowering medication, moderate alcohol use, moderate-heavy physical activity, smoking, high-school completion, family history of stroke in siblings, family history of MI in siblings, daily consumption of saturated fat, unsaturated fat, carbohydrates, and protein |
| Scrafford et al. 2010 | US | ≥17 | M/F: 14946 | 8.8 | 503 | Egg | FFQ | Men: <1 egg EO/wk  1-<7 egg EO/wk  ≥7 egg EO/wk | 1  1.26 (0.79- 2.00)  1.13 (0.61- 2.11) | Men: age, energy, marital status, race/ethnicity, BMI, diabetes, hypertension and alcohol intake  Women: age, energy, marital status, educational status, race/ethnicity, WHR, diabetes, hypertension and vitamin E. |
|  |  |  |  |  |  |  |  | Women: <1 egg EO/wk  1-<7 egg EO/wk  ≥7 egg EO/wk | 1  1.12 (0.66- 1.89)  0.92 (0.27- 3.11) |  |
| Qin et al. 2018 | China | 30–79 | M/F: 461213 | 8.9 | 9985 | Egg | FFQ | Never/rarely  1–3 days/month  1–3 days/week  4–6 days/week  7 days/week | 1  0.91 (0.85-0.98)  0.88 (0.82-0.94)  0.79 (0.73-0.87)  0.82 (0.75-0.89) | Age at recruitment, gender, education level, household income,  marital status, alcohol consumption, tobacco smoking, physical activity, BMI, waist to hip ratio, prevalent hypertension, use of aspirin, family history of CVD, intake of multivitamin supplementation and dietary pattern |
| Chen et al. 2020 | US | 50–79 | F: 96831 | 18.9 | 5589 | Dietary cholesterol | FFQ | 73.1 mg/1000 kcal/d  100 mg/1000 kcal/d  120.9 mg/1000 kcal/d  145.4 mg/1000 kcal/d  193.2 mg/1000 kcal/d | 1  1.05 (0.96-1.15)  1.02 (0.93-1.12)  1.11 (1.00-1.23)  1.19 (1.06-1.33) | Age, region, race/ethnicity, study group, education, annual family income, health insurance, smoking status, pack-years of smoking, alcohol consumption, quitting smoking/drinking owing to health problems, recreational physical activity, total energy intake, using fat to deep fry/pan fry/sauté, aspirin use, use of nonsteroidal anti-inflammatory drugs, hormone use, self-rated health status, waist circumference, diabetes, systolic and diastolic blood pressure, antihypertensive drug use, dyslipidemia, energy-adjusted fiber, saturated fat, polyunsaturated fat, monounsaturated fat, *trans* fat, animal protein, sodium |
| Sauvaget et al. 2003 | Japan | 34-103 | M/F: 40349 | 16 | 1462 | Egg | FFQ | Never  ≤1 time/week  2-4 times/week  Almost daily | 1  0.75 (0.55-1.01)  0.77 (0.57-1.03)  0.70 (0.51-0.95) | City, radiation dose, self-reported body mass index, smoking status, alcohol habits, education level, history of diabetes, or hypertension and stratified by gender, and birth cohort |
| Sauvaget et al. 2004 | Japan | 35-89 | M/F: 3731 | 14 | 60 | Dietary cholesterol | Food recall | 152 mg/d  357 mg/d  624 mg/d | 1  0.57 (0.31-1.06)  0.36 (0.17-0.76) | Age and gender stratified and adjusted for radiation dose, city, BMI, smoking status, alcohol habits, medical history of hypertension and diabetes, fruit and vegetable intake, and body weight |
| Tanasescu et al. 2004 | US | 30–55 | F:  5672 | 10 | 204 | Dietary cholesterol | FFQ | 139.6 mg/1000 kcal/d  175.9 mg/1000 kcal/d  203.6 mg/1000 kcal/d  236.5 mg/1000 kcal/d  298.2 mg/1000 kcal/d | 1  NR  NR  NR  1.35 (0.78-2.33) | Saturated fat, polyunsaturated fat, monounsaturated fat, trans fat, cholesterol, protein, calories, fiber, non-dietary covariate |
| Pietinen et al. 1997 | Finland | 50-69 | M: 21930 | 6.1 | 635 | Dietary cholesterol | FFQ | 390 mg/d  477 mg/d  543 mg/d  621 mg/d  768 mg/d | 1  0.90 (0.71-1.16)  0.81 (0.63-1.05)  0.86 (0.67-1.11)  0.92 (0.72-1.18) | Age, smoking, BMI, blood pressure, intakes of energy, alcohol, and fiber, education, and physical activity |
| Ascherio et al. 1996 | US | 40-75 | M: 43757 | 10 | 229 | Dietary cholesterol | FFQ | 189 mg/d  246 mg/d  290 mg/d  338 mg/d  422 mg/d | 1  0.92 (0.56-1.50)  1.18 (0.75-1.87)  1.11 (0.70-1.76)  1.25 (0.80-1.97) | Age, BMI, smoking habits, alcohol consumption, physical activity, history of hypertension or high blood cholesterol, family history of myocardial infarction before age 60, profession, fiber intake adjusted for energy |
| Zupo et al. 2020 | Italy | 48 | M/F:  2472 | 34 | 990 | Egg | FFQ | Per 1 egg/day | 0.73 (0.55-0.98) | Gender, (Female), Age, BMI, Education, Smoking, Comorbidity, Wine, and Olive oil |
| Wang et al. 2016 | China | 40-69 | M/F:  2445 | 26 | 355 | Egg | FFQ | Heart disease:  4 times/month | 1.00 (0.95-1.06) | age, gender, commune, smoking, drinking, season and body mass index |
|  |  |  |  |  | 452 |  |  | Stroke:  4 times/month | 1.00 (0.96-1.06) |  |
| Iribarren et al. 1996 | Japan | 59.9 | M:  6137 | 16 | 197 | Dietary cholesterol | 24-hour recall | Per 300 mg/d | 1.26(0.56-2.80) | Alcohol consumption, energy intake, BMI |
| Esrey et al. 1996 | US | 30-79 | M/F:  3954 | 12.4 | 72  40 | Dietary cholesterol | 24-hour recall | Age 30-59:  10 mg/5000 Kj | 1.00 (0.99-1.02) | Age, gender, energy intake, serum lipids, systolic blood pressure, cigarette smoking status, body mass index, and glucose intolerance. |
|  |  |  |  |  |  |  |  | Age 60-79:  10 mg/5000 Kj | 1.01 (0.98-1.03) |  |
| Misirli et al. 2012 | Greek | NR | M/F:  23601 | 10.6 | 196 | Egg | FFQ | Per 11 g/d | 1.01 (0.86-1.17) | Gender, age, education, smoking status, body mass index, level of physical activity as measured in metabolic equivalents, hypertension, diabetes, and total energy intake |

Abbreviation: RR: Relative Risk - CI: confidence interval- M: male- F: female- FFQ: food frequency questionnaire- BMI: body mass index- US: United States- NR: not-reported- wk: week

*Presented as mean or rang

**Supplemental table 4**: Characteristics of included studies on the associations between egg and cholesterol intake and cancer mortality in adults aged >18 years

| Author | Country | Age* | Sample size | Follow up (y) ^¥^ | Deaths | Exposure | Exposure assessment | Median/cutoff point | RR (95%CI) | Adjustment^†^ |
| --- | --- | --- | --- | --- | --- | --- | --- | --- | --- | --- |
| Nakamura et al. 2017 | Japan | ≥30 | F:  4686 | 15 | 210 | Egg | Food record | <1/wk  1–2/wk  1/2d  1/d  ≥2/d | 0.64 (0.33-1.26)  0.68 (0.47-0.97)  0.95 (0.68-1.32)  1  3.20 (1.51-6.76) | Age, BMI, hypertension, diabetes, cigarette smoking, alcohol drinking, dyslipidemia therapy, intake of fiber, meat, and sodium |
| Nakamura et al.2004 | Japan | ≥30 | M/F: 9263 | 14 | 356 | Egg | FFQ | Men: ≥2/d  1/d  1/2d  1-2/wk  seldom | 1.42 (0.73- 2.76)  1  1.12 (0.79- 1.58)  1.11 (0.79- 1.57)  0.60 (0.24- 1.49) | Age, serum creatinine, total cholesterol, blood glucose, BMI, SBP,DBP, use of blood pressure-lowering drugs, cigarette smoking, and alcohol intake |
|  |  |  |  |  |  |  |  | Women: ≥2/d  1/d  1/2d  1-2/wk  seldom | 2.36 (0.93- 5.98)  1  0.93 (0.61-1.41)  0.76 (0.52- 1.20)  1.18 (0.65- 2.12) |  |
| Zamora‑Ros et al. 2018 | Spain | 29-69 | M/F: 40621 | 18.4 | 1694 | Egg | Diet history | <12.6 g/d  12.6-23.35 g/d  23.35-36.5 g/d  >36.5 g/d | 1  1.07 (0.93-1.22)  1.04 (0.90-1.19)  1.11 (0.96-1.28) | Center, age at recruitment in 5 year categories, gender, smoking intensity, BMI, lifetime alcohol intake, education level, physical activity, energy intake, and adherence to Mediterranean diet |
| Sun et al. 2021 | US | 50-79 | F:  102521 | 18.1 | 7516 | Egg | FFQ | 0.03 oz./d  0.1 oz./d  0.2 oz./d  0.3 oz./d  0.7 oz./d | 1  0.97 (0.90-1.04)  1.00 (0.93-1.08)  0.95 (0.88-1.03)  1.10 (1.02-1.19) | Age at baseline, race/ethnicity, education, income, Observational Study/Clinical Trials, unopposed estrogen use, estrogen+progesterone use, smoking status, physical activity, alcohol intake, total energy intake, baseline diabetes mellitus status, baseline high blood cholesterol status, family history of heart attack/stroke, whole grain consumption, vegetable consumption, fruit consumption, sugar-sweetened beverage consumption, and mutual adjustment for other protein sources |
| Ruggiero et al. 2021 | Italy | ≥ 35 | M/F: 20562 | 8.2 | 334 | Egg | FFQ | >0≤1 n/wk  >1≤2 n/wk  >2≤4 n/wk  >4 n/wk | 1  0.98 (0.74–1.30)  1.16 (0.87–1.54)  1.52 (0.99–2.33) | Age, gender, energy intake, educational level, household income, residence, smoking, BMI, leisure-time PA, baseline diabetes, hypertension, hyperlipidemia, and Mediterranean diet score |
| Van den Brandt et al. 2019 | Netherlands | 55-69 | M/F: 7119 | 10 | 3917 | Egg | FFQ | 0 g/d  7.1 g/d  14.2 g/d  21.4 g/d | 1  0.72 (0.58-0.89)  0.78 (0.63-0.96)  0.82 (0.67-1.02) | Age at baseline, gender, cigarette smoking status, history of physician-diagnosed hypertension and diabetes, body height, BMI, non-occupational physical activity, highest level of education, intake of alcohol, vegetables and fruit, energy, use of nutritional supplements, and postmenopausal HRT |
| Farvid et al. 2017 | Iran | 36-85 | M/F: 42403 | 11 | 859 | Egg | FFQ | 0 serving /d  0.06 serving/d  0.18 serving /d  0.48 serving /d | 1  0.81 (0.67-0.99)  0.99 (0.83-1.20)  0.81 (0.67-0.99) | Age, gender, ethnicity, education, marital status, residency, smoking, opium use, alcohol, BMI, SBP, DBP, occupational physical activity, family history of cancer, wealth score, medication, energy intake |
| Kojima et al. 2004 | Japan | 40–79 | M/F: 107824 | 9.9 | 432 | Egg | FFQ | Colon cancer death  0–2/wk  3-4/wk  Everyday | 1  1.01 (0.64- 1.59)  1.17 (0.79- 1.75) | Age, family history of colorectal cancer, BMI, frequency of alcohol intake, current smoking status, walking time per day, and educational level |
|  |  |  |  |  |  |  |  | Rectal cancer death:  0–2/wk  3-4/wk  Everyday | 1  0.79 (0.38- 1.62)  0.75 (0.39- 1.46) |  |
| Wu et al. 2016 | 15 cohorts | 18-90 | M: 765852 | 9-22 | 3105 | Egg | FFQ | <5 g/d  5- <25 g/d  ≥25 g/d | 1  1.02 (0.93-1.13)  1.14 (1.00-1.30) | marital status, race, education, BMI, height (meter), alcohol, total energy intake, smoking status, prostate cancer family history, physical activity, history of diabetes, multivitamin use |
| Lin et al. 2006 | Japan | 40-79 | M/F: 110792 | 9.5 | 300 | Egg | FFQ | Men:  0-2/ month  1-4/wk  Daily | 1  1.00 (0.51-1.94)  0.81 (0.41-1.94) | Age, area, and pack-years of smoking |
|  |  |  |  |  |  |  |  | Women:  0-2/ month  1-4/wk  Daily | 1  1.56 (0.72-3.39)  1.79 (0.82-3.91) |  |
| Phillips et al. 1985 | US | >30 | M/F: 25493 | 21 | 175 | Egg | Frequency questionnaire | < 2/wk  2-4/wk  ≥5/wk | 1  1.30 (0.90-1.80)  1.50 (1.00-2.30) | Age, and gender |
| Khan et al. 2004 | Japan | >40 | M/F: 3185 | 18 | 244 | Egg | FFQ | Men:  C2 vs. C1 | 1  1.40 (0.80-2.30) | Men: Gender and smoking  Women: Health status, health education, health screening and smoking |
|  |  |  |  |  |  |  |  | Women:  C2 vs. C1 | 1  1.30 (0.70-2.50) |  |
| Sakauchi et al. 2007 | Japan | 40-79 | F: 64327 | 13.3 | 77 | Egg | FFQ | ≤1–2 times/wk  3-4 times/wk  Almost everyday | 1  0.76 (0.33-1.77)  0.65 (0.30-1.41) | Age, menopausal status, number of pregnancies, history of gender hormone use, BMI, physical activity, and education |
| Chow et al. 1992 | US | >35 | M: 17663 | 20 | 219 | Egg | FFQ | <10 times/m  10-18 times/m  19-30 times/m  >31 times/m | 1  0.90 (0.60-1.30)  1.00 (0.70-1.40)  0.90 (0.60-1.30) | Age, smoking status, and industry/occupation |
| Mills et al. 1988 | US | ≥25 | M/F: 34000 | 8 | 40 | Egg | Census and the Lifestyle Questionnaire | <1×wk  1-2×wk  ≥3×wk | 1  1.52 (0.67-3.43)  2.46 (1.08-5.63) | Age, gender |
| Zhuang et al. 2021 | US | 62.2 | M/F: 521120 | 16 | 45783 | Egg | FFQ | 0 g/2000 kcal/d  3 g/2000 kcal/d  7.5 g/2000 kcal/d  13.7 g/2000 kcal/d  28.7 g/2000 kcal/d | 1  1.05 (1.01-1.08)  1.10 (1.06-1.14)  1.10 (1.07-1.14)  1.15 (1.12-1.19) | Age, gender, BMI, race, education, marital, household income, smoking, alcohol, vigorous physical activity, usual activity at work, and history of hypertension, high cholesterol level, heart disease, stroke, diabetes, cancer at baseline, total energy, egg whites/substitutes, red meat, fish, poultry, and dairy products, fruit, vegetables, potatoes, nuts/legumes, whole grains, refine grains, coffee, and sugar-sweetened beverages |
|  |  |  |  |  |  | Dietary cholesterol |  | 118.3 mg/2000 kcal/d  168.7 mg/2000 kcal/d  207.9 mg/2000 kcal/d  252 mg/2000 kcal/d  330 mg/2000 kcal/d | 1  1.03 (0.99-1.06)  1.08 (1.05-1.12)  1.09 (1.05-1.14)  1.19 (1.14-1.24) | Age, gender, BMI, race, education, marital, household income, smoking, alcohol, vigorous physical activity, usual activity at work, and history of hypertension, high cholesterol level, heart disease, stroke, diabetes, cancer at baseline, total energy and intakes of saturated fat, polyunsaturated fat, monounsaturated fat, trans fat, animal protein, fiber, and sodium |
| Chen et al. 2020 | US | 50–79 | F: 96831 | 18.9 | 6228 | Dietary cholesterol | FFQ | 73.1 mg/1000 kcal/d  100 mg/1000 kcal/d  120.9 mg/1000 kcal/d  145.4 mg/1000 kcal/d  193.2 mg/1000 kcal/d | 1  0.94 (0.86-1.03)  0.98 (0.90-1.08)  1.03 (0.93-1.13)  1.03 (0.93-1.14) | Age, region, race/ethnicity, study group, education, annual family income, health insurance, smoking status, pack-years of smoking, alcohol consumption, quitting smoking/drinking owing to health problems, recreational physical activity, total energy intake, using fat to deep fry/pan fry/sauté, aspirin use, use of nonsteroidal anti-inflammatory drugs, hormone use, self-rated health status, waist circumference, diabetes, systolic and diastolic blood pressure, antihypertensive drug use, dyslipidemia, energy-adjusted dietary fiber, saturated fat, polyunsaturated fat, monounsaturated fat, trans fat, animal protein, sodium |
| Boeke et al. 2014 | US | 25-55 | F: 182671 | 19-30 | 1529 | Dietary cholesterol | FFQ | NR | 1  0.99 (0.82-1.20)  1.09 (0.90-1.31)  1.18 (0.98-1.42)  1.16 (0.94-1.42) | Stratified on cohort, calendar year, and age in months and adjusted for family history of breast cancer, height, age at menarche, physical activity, history of benign breast disease, birth index, lactation, BMI at 18, weight change since age 18, alcohol intake, oral contraceptive recency and duration, total energy intake, percent calories from other types of fat and protein, age at menopause, and menopausal status and postmenopausal hormone use |
| Zupo et al. 2020 | Italy | 48.00±10.71 | M/F:  2472 | 34 | 990 | Egg | FFQ | Per 1 egg/day | 0.87 (0.66-1.13) | Gender (Female), Age, BMI, Education, Smoking, Comorbidity, Wine, and Olive oil |
| Wang et al. 2016 | China | 40-69 | M/F:  2445 | 26 | 246 | Egg | FFQ | Esophageal:  4 times/month | 0.99 (0.92-1.06) | Gender, commune, smoking, drinking, season and body mass index |
|  |  |  |  |  | 175 |  |  | Gastric:  4 times/month | 1.04 (0.97-1.10) |  |
| Tokui et. 2005 | Japan | 40-79 | M: 45,181 | 11 | 574 | Egg | FFQ | 1-2/m or less  1-2/week  3-4/week  >1/day | 1  1.16 (0.79-1.69)  0.90 (0.62-1.32)  1.13 (0.79-1.62) | Age |
|  |  |  | F: 62,643 |  | 285 |  |  | 1-2/m or less  1-2/week  3-4/week  >1/day | 1  1.80 (0.91-3.53)  2.11 (1.09-4.09)  2.32 (1.22-4.42) | Age |
| Ngoan et al. 2002 | Japan  Japan | 15-96 | M: 5917 | 10 | 77 |  | FFQ | Low  Medium  High | 1  0.80 (0.40-1.50)  0.80 (0.40-1.60) | Age |
|  |  |  | F: 7333 |  | 39 |  |  | Low  Medium  High | 1  -  1.10 (0.90-1.30) | Age |

Abbreviation: RR: Relative Risk - CI: confidence interval- M: male- F: female- FFQ: food frequency questionnaire- BMI: body mass index- US: United States- NR: not-reported- wk: week

*Presented as mean or rang

**Online Supporting Material**

**Supplemental Table 5**: Results of risk of bias assessment based on the ROBINS-E tool

| Author | Bias due to confounding | Bias in selection of participants into the study | Bias in classification of exposures | Bias due to departures from intended exposures | Bias due to missing data | Bias in measurement of outcomes | Bias in selection of the reported result | Overall bias |
| --- | --- | --- | --- | --- | --- | --- | --- | --- |
| Djousse et al. 2008 | Serious | Moderate | Moderate | Low | Low | Low | Low | Serious |
| Farvid et al. 2017 | Moderate | Moderate | Moderate | Moderate | Low | Low | Low | Moderate |
| Kahn et al. 1984 | Serious | Moderate | Moderate | Moderate | Low | Low | Low | Serious |
| Zhuang et al. 2021 | Moderate | Moderate | Moderate | Moderate | Low | Low | Low | Moderate |
| Guo et al. 2017 | Moderate | Moderate | Moderate | Low | Low | Low | Low | Moderate |
| Xia et al. 2020 | Moderate | Moderate | Moderate | Moderate | Low | Low | Low | Moderate |
| Xia et al. 2020 | Serious | Moderate | Moderate | Moderate | Low | Low | Low | Serious |
| Van den Brandt et al. 2019 | Moderate | Moderate | Moderate | Moderate | Low | Low | Low | Moderate |
| Nakamura et al. 2017 | Serious | Moderate | Moderate | Moderate | Low | Low | Low | Serious |
| Nakamura et al.2004 | Serious | Moderate | Moderate | Moderate | Low | Low | Low | Serious |
| Xu et al. 2018 | Serious | Moderate | Moderate | Moderate | Low | Low | Low | Serious |
| Qureshi et al et al. 2006 | Serious | Moderate | Moderate | Moderate | Low | Low | Low | Serious |
| Mann et al. 1997 | Serious | Moderate | Moderate | Moderate | Low | Low | Low | Serious |
| Zamora‑Ros et al. 2018 | Moderate | Moderate | Moderate | Moderate | Low | Low | Low | Moderate |
| Dehghan et al. 2020 | Serious | Moderate | Moderate | Moderate | Low | Low | Low | Serious |
| Sun et al. 2021 | Moderate | Moderate | Moderate | Moderate | Low | Low | Low | Moderate |
| Ruggiero et al. 2021 | Moderate | Moderate | Moderate | Moderate | Low | Low | Low | Moderate |
| Zhong et al. 2019 | Moderate | Moderate | Moderate | Moderate | Low | Low | Low | Moderate |
| Shi et al. 2015 | Moderate | Moderate | Moderate | Moderate | Low | Low | Low | Moderate |
| Zhuang et al. 2019 | Moderate | Moderate | Moderate | Moderate | Low | Low | Low | Moderate |
| Virtanen et al. 2019 | Moderate | Moderate | Moderate | Moderate | Low | Low | Low | Moderate |
| Sauvaget et al. 2003 | Serious | Moderate | Moderate | Moderate | Low | Low | Low | Serious |
| Scrafford et al. 2010 | Moderate | Moderate | Moderate | Moderate | Low | Low | Low | Moderate |
| Qin et al. 2018 | Serious | Moderate | Moderate | Moderate | Low | Low | Low | Serious |
| Goldberg et al. 2014 | Moderate | Moderate | Moderate | Moderate | Low | Low | Low | Moderate |
| Knoops et al. 2006 | Serious | Moderate | Moderate | Moderate | Low | Low | Low | Moderate |
| Fortes et al. 2000 | Serious | Moderate | Moderate | Moderate | Low | Low | Low | Moderate |
| Chen et al. 2020 | Serious | Moderate | Moderate | Moderate | Low | Low | Low | Moderate |
| Ascherio et al. 1996 | Moderate | Moderate | Moderate | Moderate | Low | Low | Low | Moderate |
| Sauvaget et al. 2004 | Moderate | Moderate | Moderate | Moderate | Low | Low | Low | Moderate |
| Pietinen et al. 1997 | Moderate | Moderate | Moderate | Moderate | Low | Low | Low | Moderate |
| Tanasescu et al. 2004 | Moderate | Moderate | Moderate | Low | Low | Low | Low | Moderate |
| Esrey et al. 1996 | Moderate | Moderate | Moderate | Moderate | Low | Low | Low | Moderate |
| Iribarren et al. 1996 | Serious | Moderate | Moderate | Moderate | Low | Low | Low | Serious |
| Misirli et al. 2012 | Moderate | Moderate | Moderate | Moderate | Low | Low | Low | Moderate |
| Wang et al. 2016 | Serious | Moderate | Moderate | Moderate | Low | Low | Low | Serious |
| Zupo et al. 2020 | Serious | Moderate | Moderate | Moderate | Low | Low | Low | Serious |
| Trichopoulou et al. 2006 | Serious | Moderate | Moderate | Moderate | Low | Low | Low | Serious |
| Bongard et al. 2016 | Moderate | Moderate | Moderate | Moderate | Low | Low | Low | Moderate |
| Tokui et al. 2005 | Serious | Moderate | Moderate | Moderate | Low | Low | Low | Serious |
| Ngoan et al. 2002 | Serious | Moderate | Moderate | Moderate | Low | Low | Low | Serious |
| Mills et al. 1988 | Serious | Moderate | Moderate | Moderate | Low | Low | Low | Serious |
| Sakauchi et al. 2007 | Serious | Moderate | Moderate | Moderate | Low | Low | Low | Serious |
| Khan et al. 2004 | Serious | Moderate | Moderate | Moderate | Low | Low | Low | Serious |
| Phillips et al. 1985 | Serious | Moderate | Moderate | Moderate | Low | Low | Low | Serious |
| Lin et al. 2006 | Serious | Moderate | Moderate | Moderate | Low | Low | Low | Serious |
| Chow et al. 1992 | Serious | Moderate | Moderate | Moderate | Low | Low | Low | Serious |
| Wu et al. 2016 | Moderate | Moderate | Moderate | Moderate | Low | Low | Low | Moderate |
| Kojima et al. 2004 | Serious | Moderate | Moderate | Moderate | Low | Low | Low | Serious |
| Boeke et al. 2014 | Moderate | Moderate | Moderate | Low | Low | Low | Low | Moderate |
| Sluik et al. 2014 | Moderate | Moderate | Moderate | Moderate | Low | Low | Low | Moderate |

Abbreviation: ROBINS-E: risk of bias in non-randomized studies of exposures

The risk of bias in non-randomized studies of exposures (ROBINS-E) tool comprises 7 domains through which bias might be introduced. The questions of these domains include: (1) bias due to confounding, (2) bias in selection of participants into study, (3) bias in the classification of exposures, (4) bias due to departure from intended exposures, (5) bias due to missing data, (6) bias in the measurement of outcomes, and (7) bias in the selection of reported results. Studies were categorized as low risk, moderate risk, serious risk, and critical risk of bias under each domain.

**Supplemental Table 6**. Relative risks and 95% CIs from the nonlinear dose-response analysis of egg consumption and mortality

| Egg (eggs/d) | All-cause mortality | CVD mortality | Cancer mortality |
| --- | --- | --- | --- |
| 0 | 1.00 | 1.00 | 1.00 |
| 0.20 | 0.98 (0.95-1.02) | 1.01 (0.97-1.06( | 1.01 (0.97-1.06) |
| 0.40 | 0.97 (0.92-1.04) | 1.02 (0.95-1.09) | 1.04 (0.97-1.10) |
| 0.60 | 0.98 (0.91-1.06) | 1.02 (0.94-1.10) | 1.08 (1.02-1.15) |
| 0.80 | 1.01) 0.92-1.10( | 1.01 (0.93-1.10) | 1.14 (1.05-1.24) |
| 1.00 | 1.04 )0.95-1.14( | 1.01 (0.92-1.09) | 1.21 (1.05-1.39) |
| 1.20 | 1.08 )0.98-1.18( | 1.00 (0.91-1.09) | 1.28 (1.04-1.58) |
| 1.40 | 1.12 )1.02-1.23( | 0.99 (0.90-1.09) | 1.36 (1.03-1.80) |
| 1.60 | 1.16 )1.05-1.28( | 0.98 (0.88-1.09) | 1.45 (1.02-2.05) |
| 1.80 | 1.20 )1.09-1.33( | 0.97 (0.86-1.10) | 1.54 (1.01-2.33) |
| 2.00 | 1.25 )1.12-1.39( | 0.96 (0.83-1.10) | 1.63 (1.00-2.66) |
| P_-nonlinearity_ | 0.003 | 0.43 | 0.51 |

**Supplemental Table 7**. Relative risks and 95% CIs from the nonlinear dose-response analysis of dietary cholesterol intake and mortality

| Dietary cholesterol (mg/d) | All-cause mortality | CVD mortality | Cancer mortality |
| --- | --- | --- | --- |
| 50 | 1.00 | 1.00 | - |
| 100 | 0.99 (0.97-1.01) | 0.99 (0.94-1.04( | 1.00 |
| 150 | 0.97 (0.93-1.01) | 0.98 (0.88-1.09) | 1.02 (1.00-1.04) |
| 200 | 0.97 (0.91-1.03) | 0.97 (0.82-1.13) | 1.05 (0.99-1.10) |
| 250 | 0.97) 0.90-1.05( | 0.96 (0.78-1.19) | 1.08 (1.00-1.16) |
| 300 | 1.00 )0.91-1.09( | 0.98 (0.75-1.27) | 1.12 (1.03-1.22) |
| 350 | 1.04 )0.94-1.14( | 1.01 (0.74-1.38) | 1.17 (1.07-1.28) |
| 400 | 1.09 )0.97-1.21( | 1.06 (0.73-1.53) | 1.23 (1.11-1.35) |
| 450 | 1.14 )1.01-1.28( | 1.12 (0.73-1.71) | 1.28 (1.15-1.44) |
| 500 | 1.20 )1.05-1.36( | 1.18 (0.73-1.93) | 1.34 (1.18-1.53) |
| 550 | 1.26 )1.09-1.45( | 1.25 (0.72-2.17) | - |
| 600 | 1.32 (1.13-1.54) | 1.32 (0.72-2.45) | - |
| 700 | - | 1.48 (0.70-3.12) | - |
| 800 | - | 1.66 (0.69-3.99) | - |
| P_-nonlinearity_ | <0.001 | 0.009 | 0.28 |
