# Supplementary material for: Egg and Dietary Cholesterol Intake and Risk of All-Cause, Cardiovascular, and Cancer Mortality: A Systematic Review and Dose-Response Meta-Analysis of Prospective Cohort Studies
Source: Front Nutr. 2022 May 27;9:878979. doi: 10.3389/fnut.2022.878979 (PMC9195585; doi:10.3389/fnut.2022.878979)
Supplement: Supplementary file 2 [file Data_Sheet_2.docx]

**Online Supporting Material**

**Supplemental Figure 1**

Forest plot for the association between egg intake and all-cause mortality by comparing the highest and lowest intake of egg

**Online Supporting Material**

**Supplemental Figure 2**

Forest plot for the association between egg intake and all-cause mortality based on the intake of each additional egg per day

**Online Supporting Material**

**Supplemental Figure 3**

Forest plot for the association between dietary cholesterol intake and all-cause mortality by comparing the highest and lowest intake of dietary cholesterol

**Online Supporting Material**

**Supplemental Figure 4**

Forest plot for the association between dietary cholesterol intake and all-cause mortality based on a 100 mg/d increase in dietary cholesterol

**Online Supporting Material**

**Supplemental Figure 5**

Forest plot for the association between egg intake and CVD mortality by comparing the highest and lowest intake of egg

**Online Supporting Material**

**Supplemental Figure 6**

Forest plot for the association between egg intake and CVD mortality based on the intake of each additional egg per day

**Online Supporting Material**

**Supplemental Figure 7**

Forest plot for the association between dietary cholesterol intake and CVD mortality by comparing the highest and lowest intake of dietary cholesterol

**Online Supporting Material**

**Supplemental Figure 8**

Forest plot for the association between dietary cholesterol intake and CVD mortality based on a 100 mg/d increase in dietary cholesterol

**Online Supporting Material**

**Supplemental Figure 9**

Forest plot for the association between egg intake and cancer mortality by comparing the highest and lowest intake of egg

**Online Supporting Material**

**Supplemental Figure 10**

Forest plot for the association between egg intake and cancer mortality based on the intake of each additional egg per day

**Online Supporting Material**

**Supplemental Figure 11**

Forest plot for the association between dietary cholesterol intake and cancer mortality by comparing the highest and lowest intake of dietary cholesterol

**Online Supporting Material**

**Supplemental Figure 12**

Forest plot for the association between dietary cholesterol intake and cancer mortality based on a 100 mg/d increase in dietary cholesterol
